# LjCIPK1: a CBL-interacting protein kinase from *L. japonica*, confers tolerance to salt in *Arabidopsis*

**DOI:** 10.3389/fpls.2026.1828499

**Published:** 2026-04-29

**Authors:** Luyao Huang, Zhuangzhuang Li, Congzhe Hou, Qingjun Li, Gaobin Pu, Zhenhua Liu, Jia Li

**Affiliations:** 1 College of Pharmacy, Shandong University of Traditional Chinese Medicine, Shandong, China; 2 Experimental Center, Shandong University of Traditional Chinese Medicine, Shandong, China

**Keywords:** flavonoids, ion homeostasis, LjCIPK1, *Lonicera japonica*, salt stress

## Abstract

In plants, Calcineurin B-like proteins (CBLs) and their interacting kinases (CIPKs) are essential for mediating responses to various environmental stresses. This study functionally characterized *LjCIPK1* from *Lonicera japonica* and verified its role in salt stress responses. Ectopic expression of *LjCIPK1* in *Arabidopsis thaliana* significantly increased tolerance to salt stress, allowing transgenic plants to bolt, flower, and set seed normally under stress conditions. Subcellular localization assays indicated that LjCIPK1 is distributed in both the cytoplasm and nucleus, and this pattern remains unchanged under salt stress. Mechanistically, Y2H and BiFC assays revealed that LjCIPK1 interacts with the Ca^2+^ sensors LjCBL1/3/6 and the Na^+^/H^+^ antiporter LjNHX3/6/7. Overexpression of *LjNHX3* also conferred salt tolerance, supporting a role for *LjCIPK1* in maintaining intracellular ion homeostasis. In parallel, *LjCIPK1* overexpression significantly enhanced flavonoid content and upregulated key biosynthetic genes, leading to reduced ROS accumulation and oxidative damage. Furthermore, LjCIPK1 was found to interact with diverse stress regulators, including the transcription factor MYB42, protein phosphatase AIP1, glycosyltransferase UGT75B1, and kinase regulatory subunit KINB2. These results demonstrate that the LjCBL1/3/6–LjCIPK1 complex enhances salt tolerance by coordinating multiple pathways: regulating LjNHX3 to compartmentalize Na^+^ into the vacuole, activating antioxidant defense systems through flavonoid biosynthesis, and recruiting diverse downstream stress regulators to orchestrate a multifaceted stress response.

## 1 Introduction

Salinity stress ranks among the major abiotic challenges jeopardizing global agricultural sustainability. Current estimates indicate that approximately one-third of irrigated farmland worldwide is affected by elevated salinity levels (Tarolli et al., 2024; Tibesigwa et al., 2025). Given that saline-affected regions are expanding at an alarming rate due to climate change, projections suggest over half of cultivable land could face salinization by 2050 (Hassani et al., 2020; Tansley et al., 2023). As sessile organisms, plants must continually adapt to diverse environmental pressures, including high-salt conditions, by evolving complex acclimation mechanisms (Zhao et al., 2021; Zhou et al., 2024b). The discovery of salt tolerance mechanisms represents a landmark achievement in plant biology, largely driven by forward genetic screening. The identification of the *Salt Overly Sensitive* (SOS) pathway in the model plant *Arabidopsis thaliana* was a breakthrough that initiated extensive research into decoding Ca²^+^ signals via Calcineurin B-like protein (CBL)–CBL-interacting protein kinase (CIPK) modules (Liu and Zhu, 1997; Tang et al., 2020). Recent advances have further highlighted the intricate regulatory networks of these modules in orchestrating ion homeostasis (Kaya et al., 2024). Therefore, understanding Ca²^+^ signaling responses mediated by the CBL-CIPK module under salt stress is crucial for developing new approaches to breed stress-resilient crops and maintain the current global food supply in the context of increasing soil salinization (Mao et al., 2023; Liang et al., 2024).

In plants, Ca^2+^ serves dual roles as both a vital nutrient and a critical secondary messenger, participating in diverse physiological processes such as maintaining ion balance, responding to abiotic and biotic stresses, controlling stomatal aperture, guiding pollen tube elongation, and more (Feijó and Wudick, 2018). When environmental cues trigger rapid fluctuations in cytosolic Ca^2+^ levels, these changes are detected by specialized Ca^2+^-sensing proteins, which then modulate downstream signaling cascades (Jakada et al., 2025). Key Ca^2+^ sensors include calmodulin-like proteins (CMLs), calcineurin B-like proteins (CBLs), canonical calmodulins (CaMs), and calcium-dependent protein kinases (CDPKs/CPKs) (Kour et al., 2023). Upon binding Ca²^+^, these sensors undergo conformational changes that either directly modulate target proteins or activate interacting kinases. Among these, CBLs function as sensor relay proteins; they lack intrinsic kinase activity but specifically interact with and activate downstream CIPKs to transmit Ca²^+^ signals (Zhu et al., 2025). Structurally, CBLs feature four conserved EF-hand motifs—a defining characteristic that enables Ca^2+^ coordination while mediating selective interactions with NAF/FISL domains in the C-terminal regions of CIPKs (Ma et al., 2020). CIPKs, classified under the SnRK3 family, represent a unique group of plant-specific serine/threonine (Ser/Thr) kinases (Yang et al., 2022; Li et al., 2025).

Advances in plant genome sequencing have elucidated the architecture of CBL-CIPK networks across multiple species (Liu et al., 2022; Zhang et al., 2023). Within these networks, certain CBLs interact with overlapping sets of CIPKs, while some CIPKs are regulated by shared CBLs. This interplay of selective and shared protein interactions suggests a dual role—ensuring signaling precision while enabling functional cooperation within CBL-CIPK complexes (Tang et al., 2020; Li et al., 2022). Genetic studies highlight the pivotal function of this system in modulating plant adaptation to environmental stresses. Particularly under saline conditions, CBL-CIPK modules fine-tune the activity and selectivity of ion transporters (Kolukisaoglu et al., 2004). The SOS pathway—the first characterized mechanism governing ion homeostasis—comprises the Ca²^+^ sensors *AtCBL4*/*SOS3* and *AtCBL10*/*SCaBP8*, the kinase *AtCIPK24*/*SOS2*, and the Na^+^/H^+^ antiporter *AtNHX7*/*SOS1* (Tang et al., 2020). Upon salt-induced Ca²^+^ signaling, *AtCBL4*/*SOS3* recruits *AtCIPK24*/*SOS2* to the plasma membrane, activating its phosphorylation capacity. Subsequently, *AtCIPK24*/*SOS2* phosphorylates *AtNHX7*/*SOS1*, enhancing Na^+^ efflux to prevent cytotoxic accumulation (Qiu et al., 2002, 2004; Yang et al., 2019). Concurrently, *AtCIPK24* associates with tonoplast-localized *AtCBL10*, stimulating vacuolar sequestration of excess Na^+^ via *AtNHX1* (Qiu et al., 2004; Kour et al., 2023). Additionally, *AtCBL1*/*AtCBL9*-*AtCIPK23* complexes on the plasma membrane modulate K^+^ uptake through the channel *AKT1*, influencing root and stomatal K^+^ dynamics—a critical determinant of salt tolerance (Cheong et al., 2007). Given their multifunctional roles, genome-wide studies of CBL-CIPK families and salt-resistant candidate genes have been conducted in plants like rice (*Oryza sativa*) (Pandey et al., 2015), maize (*Zea mays*) (Arciniegas Vega and Melino, 2022), poplar (*Populus trichocarpa*) (Tang et al., 2010) and spinach (*Spinacia oleracea*) (Zhao et al., 2020). For example, *OsCIPK24*/*OsSOS2* interacts with *OsCBL4*/*OsSOS3* to regulate the function of *OsSOS1* on the plasma membrane and participate in the regulation of salt tolerance in rice (Pandey et al., 2015). However, it remains to be determined whether and how the CBL-CIPK network contributes to the regulation of the salt stress response in *L. japonica*.

*L. japonica* is a traditional medicinal plant in China; its dried flower buds have been prescribed in traditional Chinese medicine (TCM) to treat fever, influenza, sores, and swelling for thousands of years (Yan et al., 2017). Previous studies have shown that the species exhibits strong intrinsic salt tolerance, which is closely associated with the accumulation of bioactive components (Yan et al., 2016; Huang et al., 2019). Salt stress often induces severe oxidative damage by triggering the overproduction of reactive oxygen species (ROS). Plants counteract this toxicity by synthesizing secondary metabolites. Chlorogenic acid and luteoloside, derived from the phenylpropanoid pathway, act as potent non-enzymatic antioxidants during this process, and their accumulation significantly increases in *L. japonica* under salt stress (Yan et al., 2017, 2020). Recent evidence suggests that Ca^2+^ signaling networks can modulate secondary metabolism to enhance stress resilience (Kour et al., 2023; Kaya et al., 2024). Specific components of Ca^2+^ signaling network, such as the CBL-CIPK modules, have been directly implicated in regulating flavonoid biosynthesis to improve antioxidant capacity (Meng et al., 2021). Therefore, we hypothesized that the CBL-CIPK network might promote salt tolerance in *L. japonica* by regulating the phenylpropanoid pathway to improve ROS scavenging capacity (Yan et al., 2016, 2017, 2020). Although 17 CIPK genes were identified in *L. japonica*, indicating potential functional redundancy, *LjCIPK1* was specifically selected for this study due to its dominant upregulation under salt stress and its homology to the well-characterized ion-regulator *AtCIPK11*/*PKS5* in *Arabidopsis* (Fuglsang et al., 2007; Huang et al., 2021). Through comprehensive physiological and molecular analyses, this study functionally characterized the role of *LjCIPK1* and provides evidence supporting its contribution to improving salinity resilience in *L. japonica*.

## 2 Materials and methods

### 2.1 Plant materials and stress treatments

The salt-tolerant *L. japonica* cultivar ‘Huajin 6’ obtained from the Medicinal Botanical Garden of Shandong University of Traditional Chinese Medicine (Shandong Province, China) was used as the experimental material. Tissue samples (n = 16 plants per treatment group), including mature leaves, young leaves, stems, roots, and flowers, were collected from two-year-old potted plants in May 2021. The potted plants (n = 16 plants per treatment group) were subjected to stress treatments, including salt stress (100, 200, and 300 mM NaCl), osmotic stress (200 and 400 mM mannitol), cold stress (4°C), heat stress (45°C), and ABA treatment (100 μM ABA). Specifically, the gradient levels of NaCl and mannitol were chosen to simulate mild to severe salinity and osmotic stress, with 300 mM NaCl representing an established severe but non-lethal threshold for *L. japonica* (Yan et al., 2016; Huang et al., 2021). The temperatures of 4°C and 45°C were applied as standard parameters to induce typical chilling and acute heat shock responses, respectively (Yeh et al., 2012; Ding et al., 2019). Leaf samples for gene expression analysis were harvested at 0, 3, 6, 12, 24, 48, and 72 h after treatment, frozen immediately in liquid nitrogen, and stored at −80°C.

### 2.2 RNA extraction and quantitative real-time PCR

Total RNA was isolated from *L. japonica* leaf samples using the FastPure Plant Total RNA Isolation Kit (Vazyme, China). Subsequently, first-strand cDNA synthesis was performed with the PrimeScript RT Reagent Kit (TaKaRa, Japan), incorporating genomic DNA removal via the included gDNA Eraser. Relative gene expression levels were quantified through quantitative real-time PCR (qRT-PCR) on a CFX96 Real-Time System (Bio-Rad, USA), employing TB Green Premix Ex Taq (TaKaRa, Japan) for detection. Data analysis followed the 2^–ΔΔCt^ method, with all experimental treatments including three biological replicates and three technical replicates. Primer sequences used for amplification are detailed in **Supplementary Table 1**.

### 2.3 Cloning and bioinformatics analysis of *LjCIPK1* and its promoter

The LjCIPK1 protein sequence was retrieved from the *L. japonica* genomic database using BLASTp analysis, and its chromosomal position was determined from the same genomic resource. Sequence alignment between LjCIPK1 and *Arabidopsis* CIPKs was performed with Clustal Omega (https://www.ebi.ac.uk/services) (**Supplementary Table 2**), followed by phylogenetic tree reconstruction using MEGA 7 (Kumar et al., 2016). Structural characterization of *LjCIPK1* was carried out by examining its exon-intron architecture via the Gene Structure Display Server (GSDS, http://gsds.cbi.pku.edu.cn/) (Hu et al., 2015), with schematic illustrations generated using the Exon-Intron Graphic Maker (http://www.wormweb.org/exonintron). Multiple sequence alignment results were visualized through Jalview (Clamp et al., 2004). To identify putative regulatory elements, a 2,000 bp region upstream of the *LjCIPK1* translation start site (ATG) was scanned using PlantCARE (http://bioinformatics.psb.ugent.be/webtools/plantcare/html/) (Lescot et al., 2002).

### 2.4 Vector construction, plant transformation, and stress treatments

The complete coding sequence (CDS) of *LjCIPK1* was amplified from *L. japonica* cDNA with specific primers, and the promoter sequence of *LjCIPK1* was amplified from genomic DNA. The CDS of *LjCIPK1* was cloned into the XbaI- and SacI-digested pGFPGUSplus vector to generate the recombinant pGFPGUSplus-*LjCIPK1* construct. The CDS of *LjCIPK1* without the stop codon was cloned into the XbaI-digested pGFPGUSplus vector to generate the recombinant pGFPGUSplus-*LjCIPK1*::GFP vector for subcellular localization. The promoter sequence of *LjCIPK1* was cloned into an NcoI-digested pGFPGUSplus vector to generate the recombinant pGFPGUSplus-*ProLjCIPK1*::GUS vector. All the constructs were confirmed by sequencing.

The *Arabidopsis* Columbia-0 ecotype (Col-0) was used as a wild-type (WT) reference. Plants were grown in a growth chamber (14/10 h photoperiod, 25°C day/23°C night, 100 μmol m^−2^·s^−1^ PAR). To generate the transgenic lines, the constructed vectors were transformed into *Agrobacterium* GV3101 and then introduced into *Arabidopsis* using the floral dip method. Transgenic seeds were screened on 1/2MS medium containing 50 μg/mL kanamycin and 20 μg/mL rifampicin until homozygous transgenic lines (T3) were obtained.

The stress treatment concentrations were selected based on established *Arabidopsis* phenotypic screening protocols (Verslues et al., 2006). For agar plate assays, surface-sterilized seeds of homozygous transgenic and Col-0 plants were sown side-by-side on 10 × 10 cm square plates containing 1/2 MS medium with different concentrations of NaCl (0, 50, 100, and 150 mM) and mannitol (200 and 300 mM). The plates were vernalized at 4°C for 3 d and then transferred to the growth chamber for germination. The germination rate was recorded at 0, 12, 24, 36, 48, 60, and 72 h after transfer (n = 8 plates, 36 seeds per line per plate). For the root bending assay, 3-day-old seedlings were transferred to 150 mM NaCl plates, inverted 180°, and cultured for 3 d (n = 8 plates, 6 seedlings per line per plate). Root lengths were measured using ImageJ. Relative root elongation (RRE) was calculated as: RRE (%) = (Root length under stress/Average root length under control conditions) × 100%. For soil-based salt treatment, 20-day-old seedlings (n = 16 plants) were watered with 300 mM NaCl and photographed at 14 d and 28 d. For heat treatment, 20-day-old seedlings (n = 16 plants) were subjected to 45°C for 6 h, and the survival rate was recorded after 3 d of recovery. For soil-based assays, the Col-0 plants and three transgenic lines were grown side-by-side in the same trays to eliminate environmental variations. These specific concentrations were optimized to effectively differentiate the stress tolerance capacity between Col-0 plants and transgenic lines while avoiding basal lethality.

### 2.5 Subcellular localization and GUS staining assay

For subcellular localization, the recombinant pGFPGUSplus-*LjCIPK1*::GFP vector was transformed into *Agrobacterium* GV3101 and infiltrated into the leaves of 4-week-old *Nicotiana benthamiana* plants. After 3 d of incubation, the leaves were treated with 300 mM NaCl solution for 6 h, and GFP fluorescence was imaged using a confocal microscope.

For GUS analysis, transgenic *Arabidopsis* lines expressing *ProLjCIPK1*::GUS at different growth stages, including 12-day-old (cotyledon expansion), 18-day-old (first pair of true leaves expanded), and 30-day-old (3–4 pairs of true leaves), were selected for GUS histochemical staining. Soil-grown *Arabidopsis* seedlings were treated with 300 mM NaCl for 6 h, and histochemical staining was performed. The samples were completely immersed in GUS staining solution and incubated at 37°C for approximately 20 h until blue coloration appeared. The stained samples were decolorized in 70% ethanol for 3–5 h to remove chlorophyll until the tissues became transparent, followed by microscopic observation.

### 2.6 Yeast two-hybrid assay

The CDS of *LjCIPK1* was cloned into the EcoR I and BamH I restriction sites of the pGBKT7 (BD) vector, and the CDSs of *LjCBLs*, *LjNHXs*, and other candidate genes (MYB42, AIP1, UGT75B1, and KINB2) were cloned into the same restriction sites of the pGADT7 (AD) vector. Prior to screening, the pGBKT7-*LjCIPK1* construct was tested for auto-activation and toxicity. The pGBKT7-*LjCIPK1* and pGADT7-*LjCBLs* or pGADT7-*LjNHXs* constructs were co-transformed into the yeast strain Y2HGold using the lithium acetate method. Co-transformants were grown on SD/−Leu/−Trp (DDO) medium and were selected by growth on SD/−Leu/−Trp/−His/−Ade (QDO) medium supplemented with Aureobasidin A (AbA) and X-α-gal. pGBKT7-*LjCIPK1* was used as bait to screen for candidate interacting proteins from a cDNA library constructed from *L. japonica*. The plates were incubated at 28°C for 2–4 d.

### 2.7 Bimolecular fluorescence complementation

The CDS of *LjCIPK1* was cloned into the YFPC vector, while the CDSs of candidate interactors were cloned into the YFPN vector. These constructs were transformed into *Agrobacterium* GV3101. Suspensions of *Agrobacterium* carrying the YFPC and YFPN constructs were mixed and co-infiltrated into the leaves of 4-week-old *N. benthamiana* plants. Combinations with empty vectors were used as negative controls. YFP fluorescence was imaged using a confocal laser scanning microscope after incubation for 3 d.

### 2.8 Physiological and biochemical analysis of transgenic *Arabidopsis*

*Arabidopsis* seedlings were stained with 3,3’-diaminobenzidine (DAB) and nitroblue tetrazolium (NBT) to examine the *in situ* accumulation of H_2_O_2_ and O_2_^-^, respectively. The activities of catalase (CAT; A007-2), superoxide dismutase (SOD; A001-4), and peroxidase (POD; A084-3), as well as the contents of malondialdehyde (MDA; A003-3), proline (A107), and soluble sugar (SS; A145-1), were determined using the detection kits purchased from Nanjing Jiancheng Bioengineering Institute (China) according to the manufacturer’s instructions. qRT-PCR was performed to analyze the expression of flavonoid biosynthesis-related genes under normal conditions and salt treatment (300 mM NaCl), including *AtPAL*, *AtC4H*, *At4CL*, *AtCHS*, *AtCHI*, *AtFLS*, *AtDFR* and *AtF3H*. The total flavonoid content was measured using detection kits (Solarbio, Beijing, China). The physiological parameters mentioned above were measured after treating 20-day-old soil-grown plants (n = 16 individual plants per line/treatment) with 300 mM NaCl for 24 h.

After 15 d of 300 mM NaCl treatment, the plants (n = 16 individual plants per line/treatment) grown in pots were harvested, and the fresh weight (FW) of the whole plants, electrolyte leakage (EL) of leaves, and relative water content (RWC) of the whole plants were measured. The contents of Na^+^ and K^+^ in roots and shoots were determined using atomic absorption spectrometry.

### 2.9 Statistical analysis

All experiments were performed with at least three independent biological replicates, and the data are presented as the mean ± SD. Statistical analyses were conducted using SPSS software. Statistical significance between two groups was evaluated using Student’s t-test. For comparisons among three or more groups, a one-way analysis of variance (ANOVA) followed by Tukey’s *post-hoc* test was applied. Significance thresholds in all figures are denoted as follows: *p < 0.05, **p < 0.01, ***p < 0.001, and ****p < 0.0001.

## 3 Results

### 3.1 Sequence analysis of *LjCIPK1*

To explore the physicochemical characteristics of *LjCIPK1*, we cloned *LjCIPK1* from *L. japonica*. The CDS of *LjCIPK1* is 1,293 bp in length, corresponds to a genomic sequence of 1,400 bp, encodes a 430-amino acid polypeptide, and was mapped to chromosome 1. Phylogenetic analysis showed that the closest homolog to LjCIPK1 in *Arabidopsis* was AtCIPK11; both proteins cluster within the intron-less clade. LjCIPK1 shares 71.90% amino acid sequence similarity with AtCIPK11 in *Arabidopsis*. Consistent with typical CIPK family members, LjCIPK1 exhibits characteristic conserved domains: an N-terminal serine/threonine protein kinase catalytic (S-TKc) domain spanning residues 24–278 aa, which includes both an ATP-binding site and activation loop, coupled with a C-terminal regulatory region (304–362 aa) containing the signature NAF module for CBL interaction and a protein-protein interaction (PPI) domain (**Supplementary Figure 1**).

### 3.2 *LjCIPK1* is differentially regulated in response to various stresses and across tissue types

**Figure 1:**
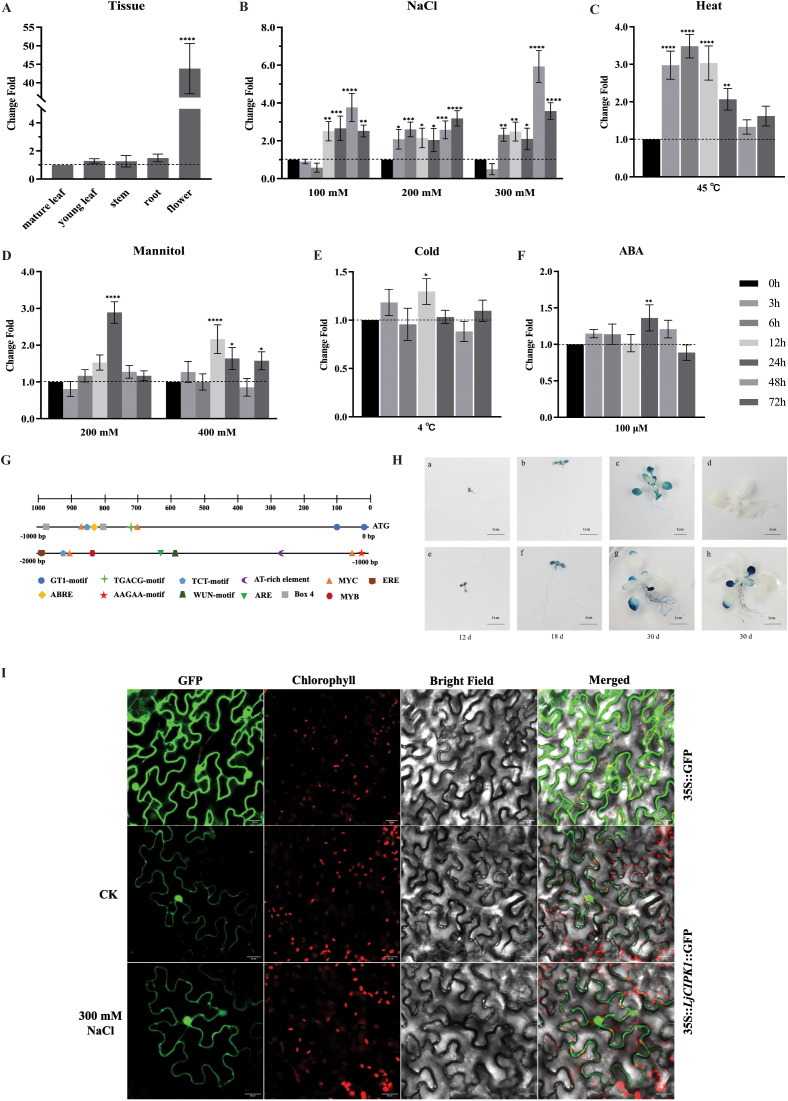
Expression profiles, subcellular localization, and promoter analysis of *LjCIPK1*. **(A)** qRT-PCR analysis of *LjCIPK1* in different tissues. The expression level in the mature leaf was set to 1.0. **(B–F)** Time-course expression profiling of *LjCIPK1* under different treatments: salt stress (100, 200, and 300 mM NaCl), osmotic stress (200 and 400 mM mannitol), cold stress (4°C), heat stress (45°C), ABA treatment (100 μM ABA). *LjActin* was used as an endogenous control. For each treatment, the expression level at 0 h was set as 1.0. Data are presented as the mean ± SD (n = 16 plants). Asterisks indicate significant differences (*p < 0.05, **p < 0.01, ***p < 0.001, ****p < 0.0001). **(G)** Analysis of *cis*-acting elements in the promoter region of *LjCIPK1*. Different shapes represent different *cis*-acting components. **(H)** GUS histochemical staining. (a–c) GUS staining of *proLjCIPK1*::GUS transgenic *Arabidopsis* at different growth stages. (d) GUS staining of Col-0 as a reference. (e–h) GUS staining of *ProLjCIPK1*::GUS transgenic *Arabidopsis* treated with salt stress (300 mM NaCl) for 6 h. **(I)** Subcellular localization of LjCIPK1 in *N. benthamiana* leaves. The panels display free GFP, LjCIPK1 under normal conditions, and LjCIPK1 under salt stress (300 mM NaCl). From left to right: GFP fluorescence, chlorophyll autofluorescence, bright field, and merged images.

To investigate the potential role of *LjCIPK1* in different tissues, its spatial expression patterns in the roots, stems, mature leaves, young leaves, and flowers of *L. japonica* were analyzed (**Figure 1A**). *LjCIPK1* was expressed in all tested tissues. The *LjCIPK1* expression level was the highest in the flower, showing a 43.80-fold increase compared with the mature leaf. The expression levels in the root, stem, mature leaf, and young leaf were similar under normal conditions.

The time-course expression profiling of *LjCIPK1* in leaves was further investigated in response to various stimuli by subjecting potted *L. japonica* to salt, mannitol, cold, heat, and ABA (**Figures 1B–F**). *LjCIPK1* was induced by salt, heat, and mannitol treatments, but there was no significant change under cold or ABA treatments. *LjCIPK1* expression was significantly upregulated by NaCl treatment, peaking at 48 h under 300 mM NaCl, increasing by 5.93-fold. For heat treatment, the expression of *LjCIPK1* increased first and then decreased with time, reaching a peak after 6 h of heat treatment, with a 3.48-fold increase. The expression of *LjCIPK1* increased to a maximum at 24 h after 200 mM mannitol treatment with a 2.89-fold increase.

### 3.3 The promoter of *LjCIPK1* is salt stress-inducible

To explore the regulatory mechanism of *LjCIPK1*, we analyzed the *cis*-acting regulatory elements in the 2,000 bp promoter region upstream of the *LjCIPK1* translation start site (ATG) using PlantCARE (**Figure 1G**). The promoter contains various *cis*-acting regulatory elements involved in stress responses and developmental processes, including GT1-motif, TGACG-motif, TCT-motif, AAGAA-motif, AT-rich element, MYC, MYB, Box 4, ABRE, ARE, and ERE.

To investigate the expression pattern driven by the *LjCIPK1* promoter, we generated *ProLjCIPK1*::GUS transgenic *Arabidopsis* plants. Under normal conditions, GUS activity was observed in all early developmental stages, including hypocotyl elongation, cotyledon maturation, and true leaf expansion. Prior to the emergence of true leaves, the GUS activity in cotyledons and hypocotyls was stronger than that in radicles (**Figures 1H, a, b**). Following the emergence of true leaves, GUS activity was detected in most true leaves, roots, and a few petioles, but the intensity was lower than that in hypocotyls and cotyledons (**Figures 1H, c**). No GUS signal was detected in Col-0 plants (**Figures 1H, d**). Notably, salt treatment induced a distinct spatiotemporal shift in expression: GUS activity increased dramatically in the roots but decreased in the true leaves compared to untreated controls (**Figures 1H, e–h**). These results indicate that the *LjCIPK1* promoter is salt-inducible and undergoes dynamic regulation in response to salt stress.

### 3.4 Subcellular localization of LjCIPK1

Understanding the subcellular localization of a protein is crucial for elucidating its function. Subcellular localization assays in *N. benthamiana* epidermal cells showed that the GFP-LjCIPK1 fusion protein was widely distributed in both the cytoplasm and the nucleus (**Figure 1I**). Under salt stress treatment, this nucleocytoplasmic localization pattern remained unchanged. To further validate this in a stable expression system, we imaged the roots of transgenic *Arabidopsis* expressing the LjCIPK1-GFP fusion protein. The fluorescence signal was similarly distributed throughout the cytoplasm and nucleus (**Supplementary Figure 2**). These consistent results confirm that LjCIPK1 functions in both compartments to coordinate stress responses.

### 3.5 Identification of LjCIPK1-interacting proteins by Y2H and BiFC

**Figure 2:**
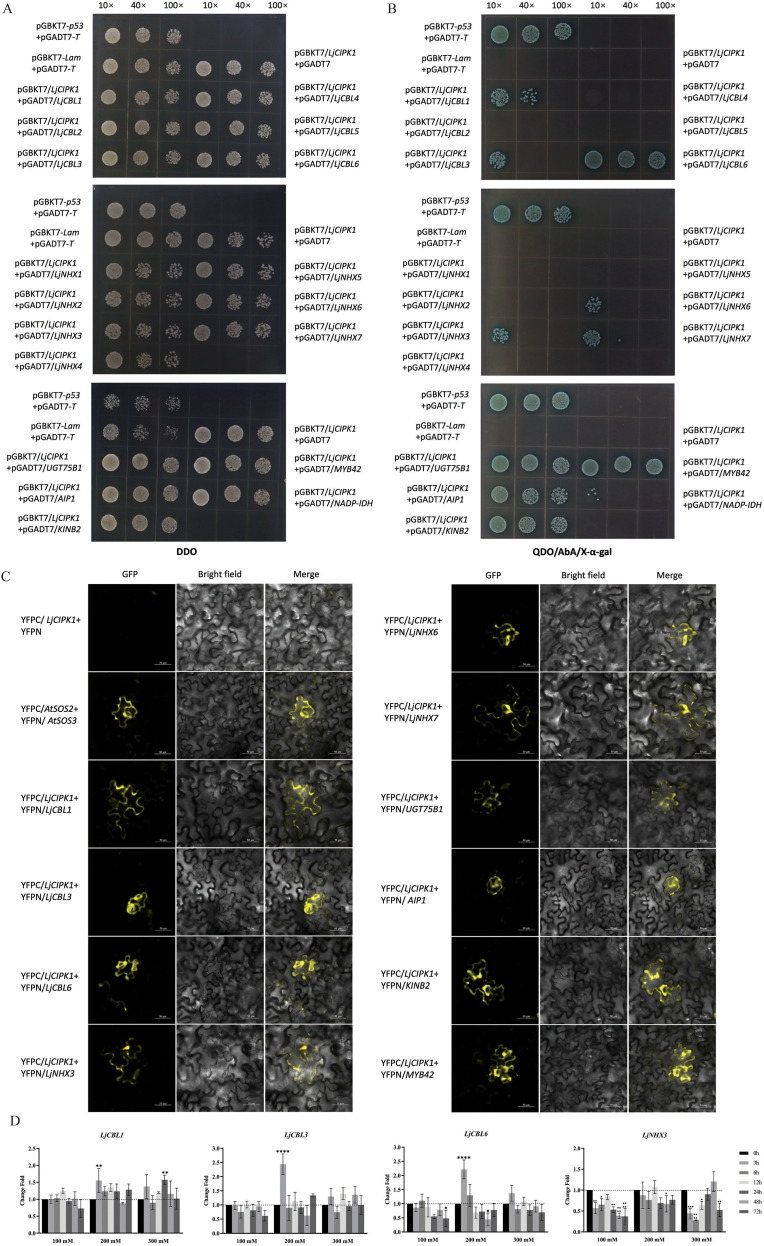
Analysis of LjCIPK1 interacting proteins. **(A, B)** Y2H assay analyzing the interaction between LjCIPK1 and candidate proteins. DDO was used to confirm the presence of both bait and prey plasmids and to evaluate the growth consistency of all co-transformants. QDO/AbA/X-α-gal was used to indicate positive physical interactions. The combination of pGADT7-T and pGBKT7–53 was used as a positive control for interaction, while the combination of pGADT7-T and pGBKT7-Lam was used as a negative control. The combination of pGBKT7-LjCIPK1 and empty pGADT7 was used to test auto-activation and toxicity. **(C)** BiFC assay of the interaction between LjCIPK1 and candidate proteins in *N. benthamiana* leaves. YFPC-AtSOS2 + YFPN-AtSOS3 was used as a positive control. **(D)** The expression profiles of *LjCBL1/3/6* and *LjNHX3* in *L. japonica* under salt stress. Data are presented as the mean ± SD (n = 16 plants). Asterisks indicate significant differences (*p < 0.05, **p < 0.01, ***p < 0.001, ****p < 0.0001).

As CIPKs are typically activated by interaction with CBLs, the interaction between LjCIPK1 and six LjCBLs identified from the *L. japonica* genome was investigated using Y2H assays. The results showed that LjCBL1, LjCBL3, and LjCBL6 physically interacted with LjCIPK1 (**Figure 2B**). CBL-CIPK complexes are known to regulate ion transporters to maintain ion homeostasis, and the most well-studied transporters regulated by CBL-CIPK modules are Na^+^/H^+^ transporter NHXs. Among the seven LjNHXs identified in the *L. japonica* genome, LjNHX3, LjNHX6, and LjNHX7 were found to interact with LjCIPK1 in Y2H assays (**Figure 2B**).

In addition, LjCIPK1 was used as a bait to screen for potential interacting proteins of LjCIPK1 from a yeast cDNA library constructed from salt-stressed *L. japonica*. The positive clones identified corresponded to 17 different proteins (**Supplementary Table 3**). The CDSs of selected candidate genes were cloned into the pGADT7 vector to verify the screening results. The results confirmed that LjCIPK1 interacted with Ljap00035735 (MYB42), Ljap00031509 (AIP1), Ljap00031268 (UGT75B1), and Ljap00012668 (KINB2) (**Figure 2B**). BiFC assays further verified the interactions between LjCIPK1 and LjCBL1/3/6, LjNHX3/6/7, and the candidate proteins (MYB42, AIP1, UGT75B1, and KINB2) in *N. benthamiana* leaves (**Figure 2C**; **Supplementary Figure 3**).

### 3.6 Salt tolerance was increased in transgenic *Arabidopsis* overexpressing *LjCIPK1*

**Figure 3:**
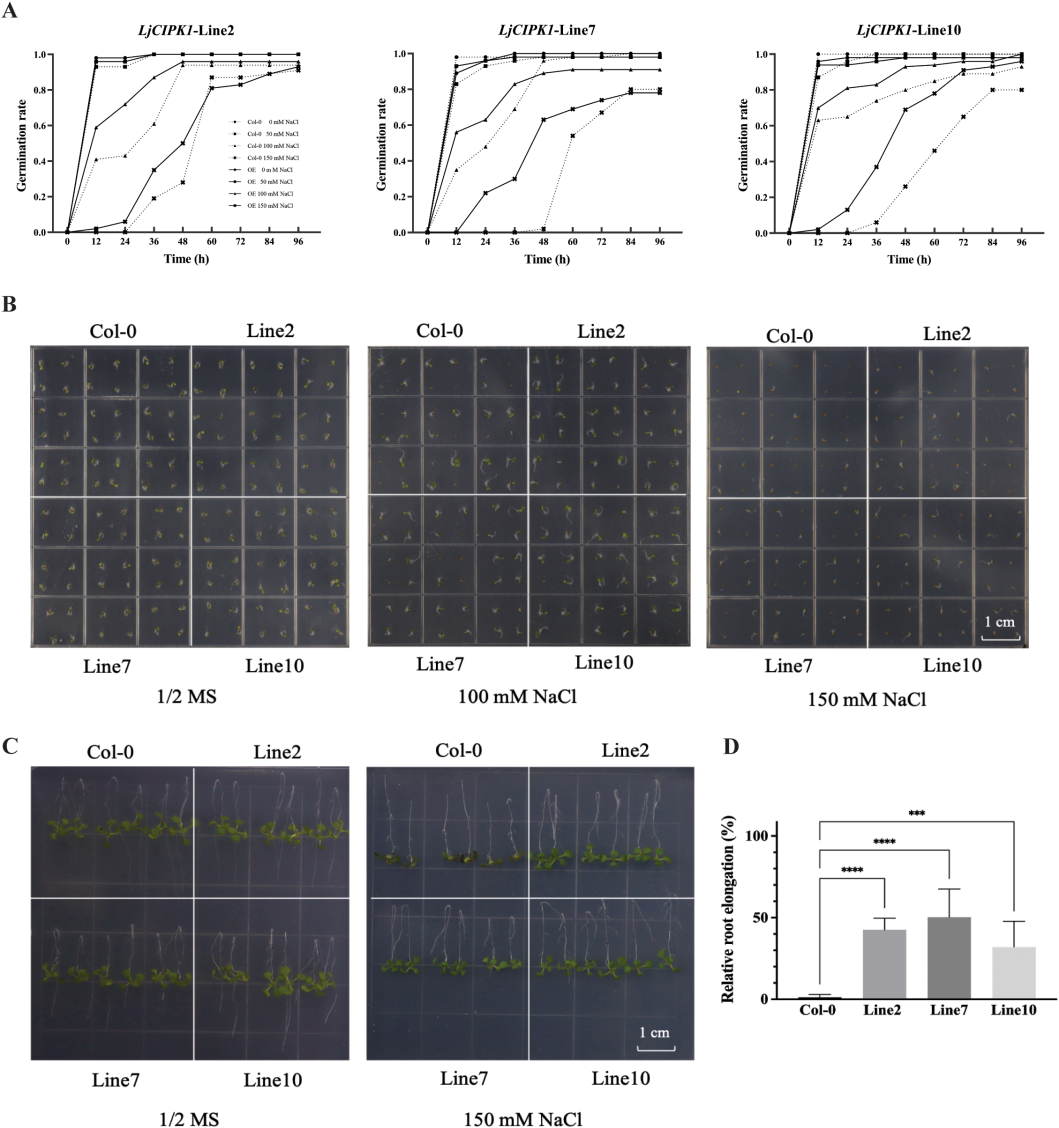
Analysis of germination rates and root length in the transgenic *Arabidopsis* overexpressing *LjCIPK1.***(A)** Statistical analysis of germination rates under 0, 50, 100, and 150 mM NaCl treatments (n = 8 plates, 36 seeds per line per plate). **(B)** Representative images of germination on 1/2 MS medium supplemented with different concentrations of NaCl. **(C)** Representative images of the root bending assay. Seedlings were transferred to 150 mM NaCl medium and inverted 180°for 3 d. **(D)** Relative root elongation calculated from the root bending assay. Data are presented as the mean ± SD (n = 8 plates, 6 seedlings per line per plate). Asterisks indicate significant differences (***p < 0.001, ****p < 0.0001).

To investigate the role of *LjCIPK1* in abiotic stress tolerance, transgenic *Arabidopsis* plants were generated. Three homozygous lines with low (Line2), medium (Line10), and high (Line7) expression levels of *LjCIPK1* were selected from 16 T3 transgenic lines for further functional study (**Supplementary Figure 4**). The transgenic and Col-0 plants showed similar germination rates on 1/2 MS medium (**Figures 3A, B**). However, under salt stress (100 and 150 mM NaCl), the germination rates of the transgenic lines were significantly higher than those of Col-0 plants. To further validate the salt tolerance phenotype, a root bending assay was performed. The results showed that upon reorientation by 180°, the transgenic lines exhibited faster root bending recovery and greater relative root elongation compared to Col-0 under 150 mM NaCl treatment (**Figures 3C, D**). However, overexpression of *LjCIPK1* did not affect the heat and mannitol tolerance of *Arabidopsis* (**Supplementary Figure 5**, **6**).

**Figure 4:**
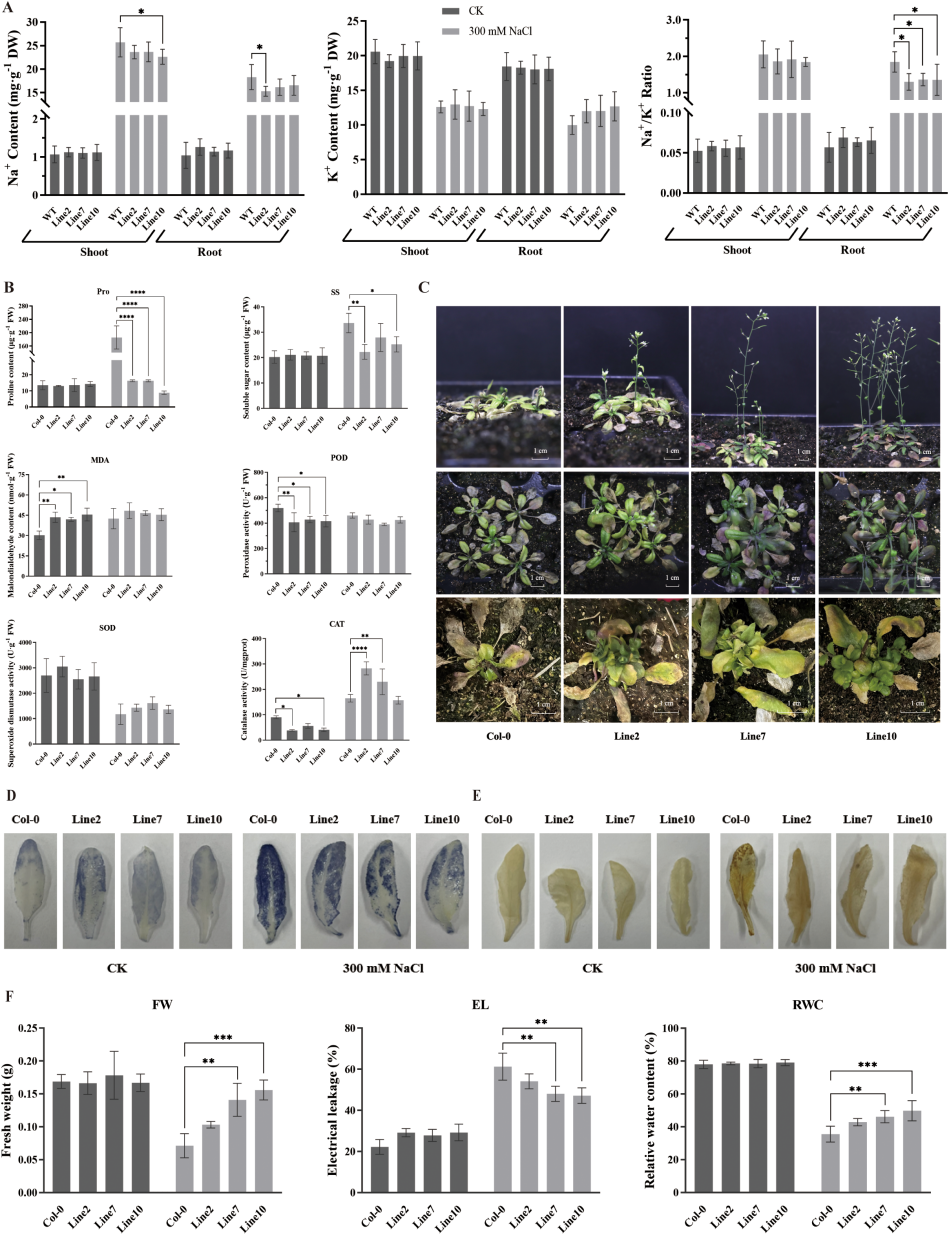
Overexpression of *LjCIPK1* in *Arabidopsis* enhances salt tolerance. **(A)** Analysis of Na^+^, K^+^ content, and the Na^+^/K^+^ ratio in roots and shoots. **(B)** Analysis of proline (Pro), soluble sugar (SS), malondialdehyde (MDA), peroxidase (POD), superoxide dismutase (SOD) and catalase (CAT) contents and activities. **(C)** Phenotypes of transgenic *Arabidopsis* and Col-0 plants under salt stress. Top and middle panels: plants treated with 300 mM NaCl for 14 days. Bottom panel: recovery of plants 14 days after the removal of salt stress. **(D)** O_2_^-^ visualization through NBT staining. **(E)** H_2_O_2_ detection via DAB staining. **(F)** Analysis of fresh weight (FW), electrolyte leakage (EL), and relative water content (RWC). Data are presented as the mean ± SD (n = 16 plants). Asterisks indicate significant differences (*p < 0.05, **p < 0.01, ***p < 0.001, ****p < 0.0001).

To test whether the overexpression of *LjCIPK1* affects reactive oxygen species (ROS) production, 20-day-old soil-grown seedlings were treated with 300 mM NaCl for 24 h. Under normal conditions, the transgenic lines exhibited higher MDA content and lower POD and CAT activities compared to Col-0. However, following salt treatment, while the MDA content in Col-0 increased significantly, no significant changes were observed in the transgenic lines, suggesting that the transgenic plants experienced less salt-induced oxidative damage. Salt stress induced CAT activity in both genotypes, but the increase was more pronounced in the transgenic lines (**Figure 4B**). Consistent with these findings, DAB and NBT staining results indicated that the accumulation and distribution of H_2_O_2_ and O_2_^-^ were lower in the cells of transgenic lines compared to Col-0 (**Figures 4D, E**). Regarding osmotic adjustment, while the proline (Pro) and soluble sugar (SS) contents increased significantly in Col-0 under salt stress, no significant changes were detected in the transgenic lines.

To evaluate salt tolerance at the adult stage, 20-day-old soil-grown seedlings were treated with 300 mM NaCl for 14 days. During the stress period, both the Col-0 and transgenic plants exhibited growth reduction, characterized by the gradual yellowing and withering of the outer rosette leaves. Despite this stress, both genotypes initiated bolting. However, a critical difference emerged during the subsequent reproductive stage: while the Col-0 flowers soon withered and desiccated, preventing them from setting seeds, the LjCIPK1-overexpressing lines maintained reproductive development, successfully flowering and producing viable seeds. Furthermore, after the removal of salt stress, the transgenic plants quickly resumed active growth, generating new rosette leaves, whereas the Col-0 plants completely collapsed (**Figure 4C**). Physiological analysis showed that transgenic lines maintained higher fresh weight (FW) and relative water content (RWC) and exhibited lower electrolyte leakage (EL) compared to Col-0 (**Figure 4F**). Furthermore, although salt stress increased the Na^+^ content in all plants, the accumulation was significantly higher in Col-0. Consequently, the Na^+^/K^+^ ratio in the roots of transgenic lines was significantly lower than that of Col-0 plants under salt stress (**Figure 4A**). These results demonstrate that *LjCIPK1* confers salt tolerance by reducing Na^+^ accumulation and enhancing ROS scavenging.

### 3.7 Overexpression of *LjCIPK1* alters the expression profiles of key genes in the flavonoid biosynthetic pathway

**Figure 5:**
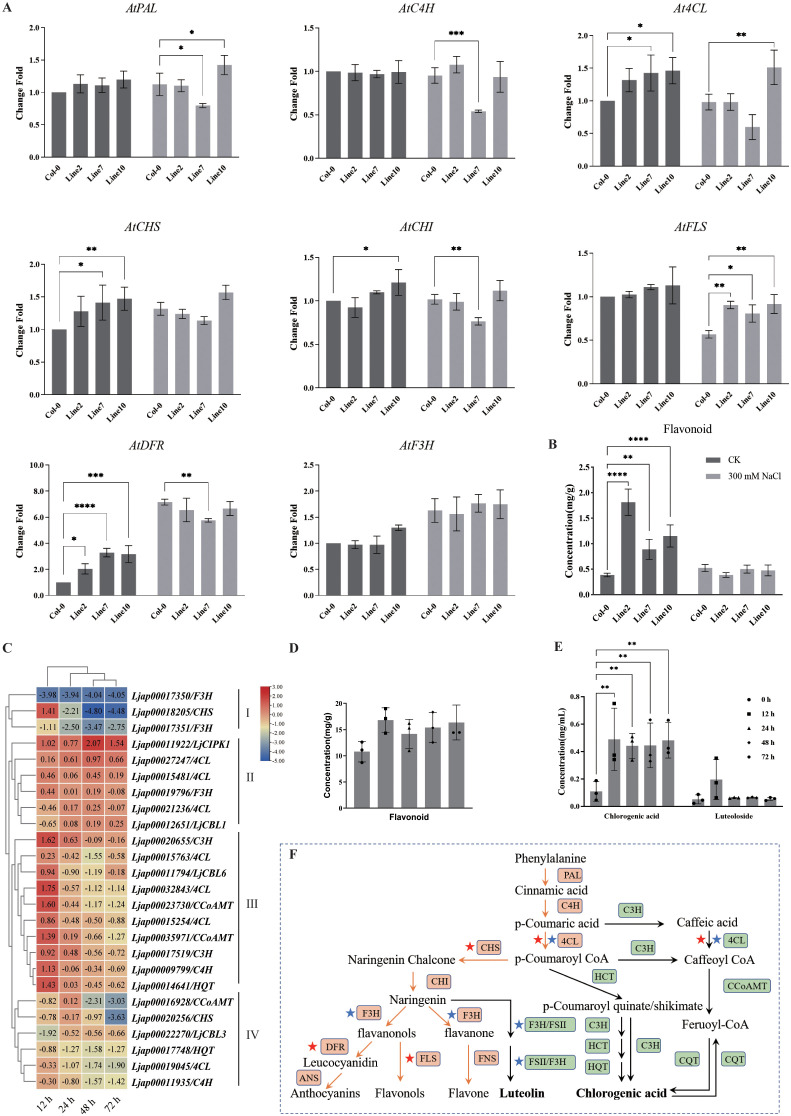
Overexpression of *LjCIPK1* promotes flavonoid biosynthesis. **(A)** Relative expression levels of key regulatory genes in the flavonoid biosynthetic pathway in transgenic *Arabidopsis* under salt stress. **(B)** Total flavonoid content in transgenic *Arabidopsis* under salt stress. **(C)** Co-expression analysis of *LjCIPK1* and key regulatory genes in the flavonoid biosynthetic pathway in *L. japonica*. The data were derived from transcriptome profiling of *L. japonica* treated with 300 mM NaCl for 12, 24, 48, and 72 h. **(D)** Total flavonoid content in *L. japonica* under salt stress. **(E)** Contents of chlorogenic acid and luteoloside in *L. japonica* under salt stress. **(F)** Schematic diagram of the phenylpropanoid biosynthetic pathway in *L. japonica* and *Arabidopsis*. Green indicates *L. japonica*; Yellow indicates *A. thaliana*. Data are presented as the mean ± SD (n = 16 plants). Asterisks indicate significant differences (*p < 0.05, **p < 0.01, ***p < 0.001, ****p < 0.0001).

Under salt stress, the contents of total flavonoids and chlorogenic acid in *L. japonica* were significantly increased, with luteoloside levels showing a distinct elevation after 12 h of treatment (**Figures 5D, E**). Furthermore, transcriptome data analysis revealed that *LjCIPK1* is co-expressed with *LjCBL1*, *4CL*, and *F3H* in *L. japonica* under salt stress (**Figure 5C**).

To validate the regulatory role of *LjCIPK1* in this pathway, we further analyzed the transgenic *Arabidopsis* lines. The total flavonoid content in transgenic *Arabidopsis* was significantly higher than that in Col-0 plants (**Figure 5B**). Consistent with this, the expression levels of *At4CL*, *AtCHS*, and *AtDFR*, which are key enzyme genes in the flavonoid biosynthetic pathway, were significantly upregulated. Under salt stress, the expression of *AtDFR* increased significantly in both Col-0 and transgenic plants. In contrast, while the expression of *AtFLS* generally decreased under salt stress, its expression level in transgenic *Arabidopsis* remained significantly higher than in Col-0 plants (**Figure 5A**). These findings indicate that LjCIPK1 promotes flavonoid accumulation by activating these downstream biosynthetic genes, thereby mitigating ROS-induced oxidative damage and enhancing salt tolerance.

### 3.8 Overexpression of *LjNHX3*, but not *LjCBL1*/*3*/*6*, enhances salt tolerance in transgenic *Arabidopsis*

**Figure 6:**
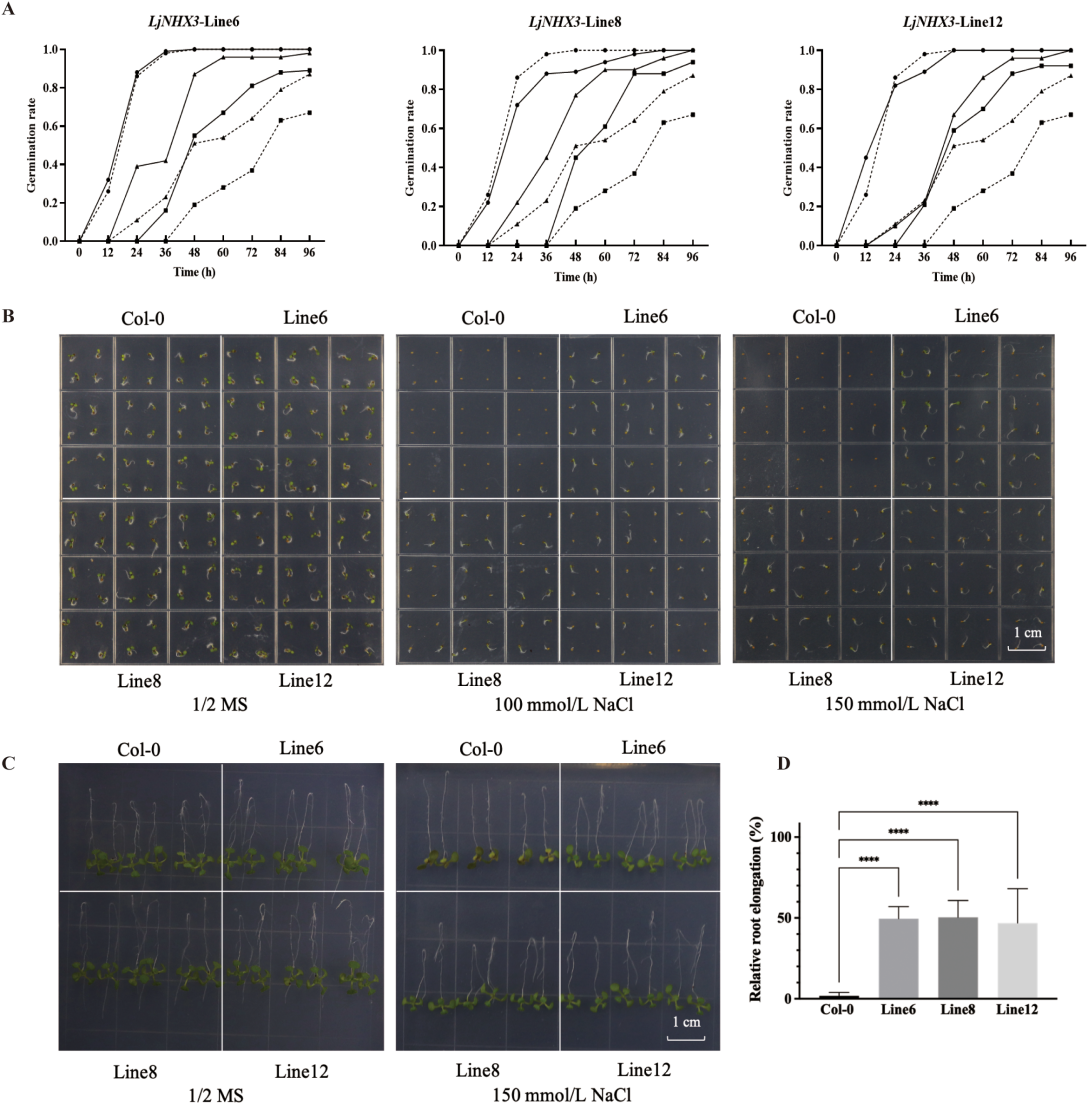
Overexpression of *LjNHX3* in *Arabidopsis* enhances salt tolerance. **(A, B)** Germination rates of transgenic *Arabidopsis* and Col-0 plants treated with 100 mM and 150 mM NaCl for 12, 24, 36, 48, 60, 72, and 96 h (n = 8 plates, 36 seeds per line per plate). **(C)** Representative images of the root bending assay. Seedlings were transferred to 150 mM NaCl medium and inverted 180°for 3 d. **(D)** Relative root elongation calculated from the root bending assay. Data are presented as the mean ± SD (n = 8 plates, 6 seedlings per line per plate). Asterisks indicate significant differences (****p < 0.0001).

To investigate the functions of *LjCBL1*/*3*/*6* and *LjNHX3* in the salt tolerance of plants, transgenic *Arabidopsis* plants were generated using the floral dip method. Three homozygous lines with low, medium, and high gene expression were selected from 16 T3 transgenic lines for further functional analysis. Under normal conditions, the transgenic and Col-0 plants showed similar germination rates and root lengths on 1/2 MS medium. Under salt stress, the germination rates of transgenic *Arabidopsis* overexpressing *LjCBL1*, *LjCBL3*, or *LjCBL6* did not differ significantly from those of Col-0 plants (**Supplementary Figure 7**). In contrast, the germination rates of *Arabidopsis* overexpressing *LjNHX3* were significantly higher than those of Col-0 plants under salt stress (**Figures 6A, B**). The transgenic lines exhibited faster root bending recovery and greater relative root elongation compared to Col-0 under 150 mM NaCl treatment (**Figures 6C, D**). qRT-PCR results showed that the expression of *LjNHX3* was downregulated by salt stress, while the expression of *LjCBL1*/*3*/*6* showed no significant changes (**Figure 2D**).

## 4 Discussion

The activation of Ca^2+^ signaling cascades plays a pivotal role in enabling plants to perceive and adapt to environmental fluctuations (Perez-Alonso et al., 2022). As major Ca^2+^ sensors, CBLs and their interacting partners, CIPKs, constitute a sophisticated network that decodes Ca^2+^ signatures in response to abiotic and biotic stresses. While the functional versatility of CBL-CIPK modules has been extensively characterized in model species, the specific regulatory mechanisms within the traditional medicinal plant *L. japonica* remain to be fully elucidated. A total of 17 CIPK genes were identified in *L. japonica* (Huang et al., 2021), potentially providing functional redundancy (Tang et al., 2020); however, distinct expression patterns often determine specific primary roles. *LjCIPK1* exhibited the most significant and rapid upregulation under salt stress among all members, indicating a dominant and non-redundant function in early stress signaling. Furthermore, its close homology to the *Arabidopsis* ion homeostasis regulator *PKS5* solidified *LjCIPK1* as the primary candidate for investigating salt tolerance mechanisms (Fuglsang et al., 2007). In this study, we demonstrated that the regulatory network centered on *LjCIPK1* is a key determinant of salt tolerance in *L. japonica*, highlighting *LjCIPK1* as a promising candidate gene for enhancing plant salinity resistance.

CIPKs play a central regulatory role in plant stress responses. Extensive studies in *Arabidopsis* have demonstrated that specific CBL-CIPK modules govern ion homeostasis. For instance, *AtCIPK24*/*SOS2*, activated by *AtCBL4*/*SOS3* or *AtCBL10*/*SCaBP8*, phosphorylates and activates the plasma membrane Na^+^/H^+^ antiporter *AtSOS1* to extrude excess Na^+^ (Huertas et al., 2012; Plasencia et al., 2020). Similarly, *AtCIPK6* and *AtCIPK8* have been implicated in modulating responses to salt and osmotic stresses. Yin et al. found that *AtCBL10*-*AtCIPK8*-*AtSOS1* functions in *Arabidopsis* to regulate salt tolerance (Yin et al., 2020). *AtCIPK6* responds to multiple stresses, negatively regulating the immune response to pathogenic microorganisms while positively regulating non-biological stresses such as salt and drought (Tsou et al., 2012; Chen et al., 2013; Gutierrez-Beltran et al., 2017; Sardar et al., 2017). Consistent with the function of these homologs, our research established that *LjCIPK1* is significantly upregulated in response to salt stress. Phenotypic analysis showed that overexpression of *LjCIPK1* in *Arabidopsis* markedly enhanced salt tolerance. Notably, regardless of the varied expression levels among the transgenic lines, ectopic expression of *LjCIPK1* significantly increased germination rates, root elongation, and overall salinity resilience compared to the wild type. Physiologically, *LjCIPK1* transgenic lines exhibited enhanced ROS-scavenging capacity and reduced oxidative damage, underscoring the role of LjCIPK1 as a robust positive regulator of salt tolerance in *L. japonica*.

Promoters play an essential role in precisely regulating gene expression (Ma et al., 2021; Shi et al., 2021). GUS staining assays revealed that salt stress induced spatiotemporal changes in *LjCIPK1* expression, leading to a marked increase in the roots. Regarding subcellular distribution, unlike the *Arabidopsis* SOS2 kinase which is recruited to the plasma membrane (Quintero et al., 2002), our localization assays indicated that LjCIPK1 is localized in both the cytoplasm and the nucleus under normal and salt stress conditions. Although CIPKs are typically known for regulating membrane transporters, several studies have implicated them in nuclear signaling events. For instance, AtCIPK1 localizes to both the cell membrane and the nucleus (D’Angelo et al., 2010), and AtCIPK3 has been shown to interact with nuclear transcription factors to modulate stress responses (Sanyal et al., 2017). In our study, the dual localization of LjCIPK1 in both *N. benthamiana* epidermal cells and transgenic *Arabidopsis* roots suggests that it functions in multiple cellular compartments. Complementing GUS staining results which demonstrated strong root tissue expression, the subcellular imaging confirms the presence of LjCIPK1 within the root cell cytoplasm and nucleus. The nuclear pool of LjCIPK1 may be involved in modulating the transcription of stress-responsive genes, which aligns with the observed upregulation of flavonoid biosynthetic genes and the subsequent accumulation of flavonoids in the transgenic plants. Simultaneously, the cytoplasmic pool of LjCIPK1 likely targets LjNHX3 at the vacuolar membrane to facilitate ion sequestration.

Although LjCBL1/3/6 were identified as physical interactors of LjCIPK1, their overexpression in *Arabidopsis* did not significantly enhance salt tolerance. This observation highlights the functional complexity of the CBL-CIPK signaling network. As Ca^2+^ sensors, CBLs do not possess enzymatic activity themselves but function by recruiting and activating specific downstream kinases. Therefore, increasing the abundance of the sensor alone may be insufficient to amplify the signal output without a concurrent increase in the active kinase partner. Furthermore, given the high specificity of CBL-CIPK interactions, ectopic LjCBLs may require specific kinase partners from *L. japonica* to function optimally, suggesting that the efficacy of CBL-mediated salt tolerance largely depends on the availability of its cognate CIPK partners.

The ion toxicity caused by high salinity is alleviated by the adjustment of cellular Na^+^ contents and Na^+^/K^+^ homeostasis through the functions of ion transporters (Wang et al., 2021). It has been well established that the *SOS3*-*SOS2*-*SOS1* (*CBL4*-*CIPK24*-*NHX7*) module regulates intracellular Na^+^ efflux under salt stress, while the *CBL1*/*CBL9*-*CIPK23*-*AKT1* module regulates K^+^ uptake (Sanyal et al., 2015; Sanchez-Barrena et al., 2020; Zhang et al., 2020). The critical role of the CBL-CIPK signaling pathway in maintaining ion homeostasis has been validated in multiple species, including rice (*Oryza sativa*) (Kanwar et al., 2014), maize (*Zea mays*) (Arciniegas Vega and Melino, 2022), and cotton (*Gossypium hirsutum*) (Su et al., 2020). In triploid bermudagrass, the *CdtCBL4*-*CdtCIPK5* pathway confers salinity tolerance by maintaining Na^+^/K^+^ homeostasis (Huang et al., 2020). Both Na^+^ efflux and vacuolar compartmentalization rely on the membrane transport activity of NHXs. In *Arabidopsis*, eight AtNHX genes have been identified, some of which confer salt tolerance via diverse mechanisms (Cui et al., 2020). In *L. japonica*, we identified an interaction between *LjCIPK1*, a positive regulator of salt tolerance, and *LjNHX3*/*6*/*7*, which are putative Na^+^/H^+^ antiporters. In this study, we focused on *LjNHX3* to further elucidate the ion transport mechanism. In our previous work, we identified *LjNHX3* as a member of the vacuolar NHX subfamily and an ortholog of *Arabidopsis AtNHX2* (*At3g05030*) (Huang et al., 2022). *AtNHX2* is a classic vacuolar transporter that mediates the exchange of H^+^ for cations (Na^+^ or K^+^) across the tonoplast, playing a critical role in vacuolar ion compartmentalization (Yokoi et al., 2002). Therefore, *LjNHX3* likely functions as a vacuolar Na^+^/H^+^ antiporter. Collectively, the physical interaction between *LjCIPK1* and *LjNHX3* suggests that *LjCIPK1* enhances salt tolerance by activating *LjNHX3* to sequester excess cytoplasmic Na^+^ into the vacuole. Furthermore, considering the interaction observed between LjCIPK1 and LjNHX6/7, it is plausible that LjCIPK1 also modulates Na^+^ efflux or intracellular trafficking pathways, collectively contributing to the improved ion homeostasis and reduced Na^+^ accumulation in transgenic plants (Huang et al., 2022).

Beyond the regulation of ion homeostasis, our study demonstrated that LjCIPK1 plays a crucial role in maintaining cellular redox balance. ROS scavenging is a vital protective mechanism under salt stress. We observed that *LjCIPK1* overexpression significantly enhanced the activities of antioxidant enzymes and reduced ROS accumulation. Importantly, this enhanced antioxidant capacity was accompanied by the upregulation of key flavonoid biosynthetic genes and the accumulation of flavonoids, which are potent non-enzymatic antioxidants.

Recently, the diverse mechanisms by which the CIPK gene family regulates plant resistance have been continuously reported. In rice (*Oryza sativa*), *OsCIPK17* interacts with *OsPP2C77*, *OsJAMYB*, *OsNAC77*, and *OsDREB1H* to regulate growth and stress resistance (Gao et al., 2025). In this study, we predicted and verified target proteins that may have a regulatory relationship with *LjCIPK1*, including Ljap00035735 (Transcription repressor *MYB42*), Ljap00031509 (Protein phosphatase 2C 3, *AIP1*), Ljap00031268 (UDP-glycosyltransferase 75B1, *UGT75B1*), and Ljap00012668 (SNF1-related protein kinase regulatory subunit beta-2, *KINB2*).

Ljap00031268 is annotated as a glycosyltransferase (GT). GTs catalyze the glycosylation of flavonoids, terpenes, sterols, alkaloids, phenols, hormones, sugars, proteins, and lipids, thereby participating in plant secondary metabolic pathways, detoxification, and responses to biotic and abiotic stresses. In rice (*Oryza sativa*), 40 GT8 genes have been identified; notably, *OsGolS1* was upregulated under salt stress, while *OsGAUT21*, *OsGATL2*, and *OsGATL5* exhibited significant upregulation under cold stress (Kong et al., 2019). Similarly, *SlGolS1* positively regulates cold resistance in tomato (Zhang et al., 2024), and the *ZmHSF08*-*ZmUGT92A1* module regulates heat tolerance by altering ROS levels in maize (Li et al., 2024). However, how CIPKs interact with GTs to regulate resistance remains largely unknown.

Ljap00031509 is annotated as a type 2C protein phosphatase (PP2C), which participates in plant adaptation to stresses such as saline-alkali, drought, and low temperature by affecting various metabolic processes and hormone regulation (Sung et al., 2007; Chuong et al., 2021). In *Arabidopsis*, the CBL-CIPK family forms a phosphorylation/dephosphorylation network with PP2Cs, regulating the K^+^ transport channel AKT1 (Lan et al., 2011). In addition, *ABI1*, *ABI2*, and *AIP1*, members of the PP2C family, can interact with CIPKs to regulate downstream proteins (Ohta et al., 2003; Tang et al., 2020).

Ljap00012668 is annotated as a regulatory subunit beta-2 of SNF1-related protein kinase (SnRK), which plays a role in signal transduction cascades regulating gene expression and carbohydrate metabolism (Lovas et al., 2003). Among the ten SnRK2 family members in *Arabidopsis* (Hrabak et al., 2003; Mao et al., 2019), six are inducible by ABA, and nine can be activated by high osmotic pressure induced by salt stress (Boudsocq et al., 2004). *AtSnRK2.4* and *AtSnRK2.10* regulate root system architecture under salt stress by influencing the expression of aquaporin genes (Dorota et al., 2019). Yan et al. found that *CIPK14* interacts with three CBLs and two key kinases, *SnRK1.1* and *SnRK1.2*, which are implicated in glucose signaling (Yan et al., 2014).

Ljap00035735 is annotated as a MYB transcription factor. The MYB family is one of the largest transcription factor families in plants and is widely involved in cell morphogenesis, secondary metabolite biosynthesis, meristem formation, and resistance to biotic and abiotic stresses (Hu et al., 2024; Zhou et al., 2024a). Yu et al. provided evidence that *VaCIPK18* functions as a key node in the CBL-CIPK network and may interact with *VaMYB4a* to contribute to the cold response in grape plants (Yu et al., 2022). Therefore, the ‘CIPK1–MYB42/AIP1/UGT75B1/KINB2’ module may represent a novel mechanism for regulating salt tolerance. The in-depth exploration of the functions of these genes will expand our understanding of the mechanisms by which plants respond to salt stress.

## 5 Conclusions

**Figure 7:**
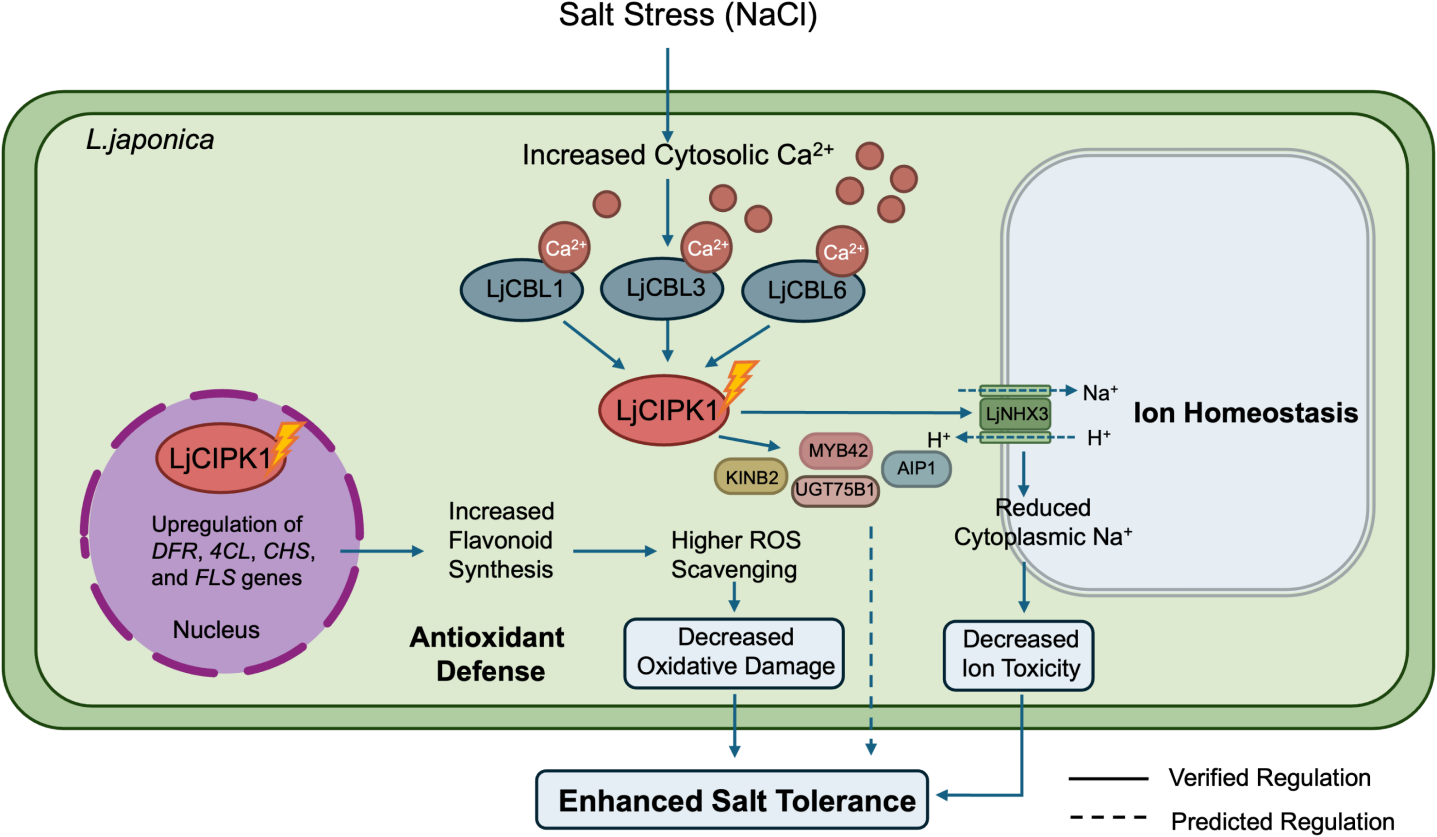
A proposed model for *LjCIPK1*-mediated salt tolerance in *L. japonica*. Salt stress triggers cytosolic Ca²^+^ signaling, which is decoded by LjCBL1/3/6 to recruit and activate LjCIPK1. The activated LjCIPK1 functions in both the cytoplasm and the nucleus to regulate parallel pathways: (1) LjCIPK1 activates the putative vacuolar transporter LjNHX3, promoting Na^+^ sequestration into the vacuole to reduce cytoplasmic Na^+^ toxicity. (2) LjCIPK1 interacts with diverse stress regulators, including MYB42, AIP1, UGT75B1, and KINB2 to enhance salt tolerance. (3) LjCIPK1 promotes the expression of flavonoid biosynthetic genes, leading to accumulated flavonoids and enhanced ROS scavenging.

This study characterizes *LjCIPK1* as a positive regulator of salt tolerance in *L. japonica*. Our findings indicate that cytoplasmic and nuclear LjCIPK1, activated by LjCBL1/3/6, confers stress resistance by physically interacting with the vacuolar transporter *LjNHX3* to facilitate Na^+^ sequestration, while synergistically mitigating oxidative damage by promoting flavonoid biosynthesis to enhance ROS scavenging. Furthermore, the identification of novel interactors, including MYB42, AIP1, UGT75B1, and KINB2, suggests that LjCIPK1 functions as a signaling hub that recruits diverse stress regulatory factors to orchestrate a complex and multifaceted response to salinity (**Figure 7**). These insights not only deepen our understanding of the CBL-CIPK network in woody medicinal plants but also highlight *LjCIPK1* as a promising candidate for engineering salt-tolerant crops.

## Data Availability

The datasets generated during the current study are available at NCBI GenBank: MZ796249–MZ796262. The accession numbers of the genes used in this study are as follows: *LjCBL1* (Ljap00012651), *LjCBL2* (Ljap00029480), *LjCBL3* (Ljap00022270), *LjCBL4* (Ljap00010024), *LjCBL5* (Ljap00010536), *LjCBL6* (Ljap00011794), *LjCIPK1* (Ljap00011922), *LjNHX1* (Ljap00034208), *LjNHX2* (Ljap00014455), *LjNHX3* (Ljap00006626), *LjNHX4* (Ljap00011905), *LjNHX5* (Ljap00015571), *LjNHX6* (Ljap00035581), *LjNHX7* (Ljap00009930).

## Supplementary material

Supplementary Figure 1 Identification of *LjCIPK1* in *L. japonica.*

Supplementary Figure 2 Subcellular localization of LjCIPK1 in transgenic *Arabidopsis* roots.

Supplementary Figure 3 BiFC negative control assays for LjCIPK1-interacting partners. No YFP fluorescence signal was observed in any combination, confirming that the fluorescence signals observed in **Figure 2E** were due to specific protein-protein interactions.

Supplementary Figure 4 qRT-PCR analysis of *LjCIPK1* expression in transgenic *Arabidopsis* lines. Relative expression levels of *LjCIPK1* were measured in 16 independent transgenic lines. Lines 2, 10, and 7 were selected as representative lines with low, medium, and high expression levels, respectively, for further study.

Supplementary Figure 5 Germination rates of transgenic *Arabidopsis* overexpressing *LjCIPK1* under mannitol stress.

Supplementary Figure 6 Survival rates of transgenic *Arabidopsis* overexpressing *LjCIPK1* under heat stress.

Supplementary Figure 7 Germination rates of transgenic *Arabidopsis* overexpressing *LjCBL1*/*3*/*6* under salt stress.

Supplementary Table 1 Primer sequences used in the study.

Supplementary Table 2 Protein sequences of LjCIPK1 and CIPKs from *Arabidopsis*.

Supplementary Table 3 Seventeen potential interacting proteins of LjCIPK1 from a yeast cDNA library.
