# Supplementary material for: LjCIPK1: a CBL-interacting protein kinase from L. japonica, confers tolerance to salt in Arabidopsis
Source: Front Plant Sci. 2026 Apr 29;17:1828499. doi: 10.3389/fpls.2026.1828499 (PMC13167526; doi:10.3389/fpls.2026.1828499)
Supplement: Supplementary Figure 1 — Identification of LjCIPK1 in L. japonica. [file DataSheet1.docx]

Supplementary Material

# Supplementary Figures and Tables

## Supplementary Figures

**
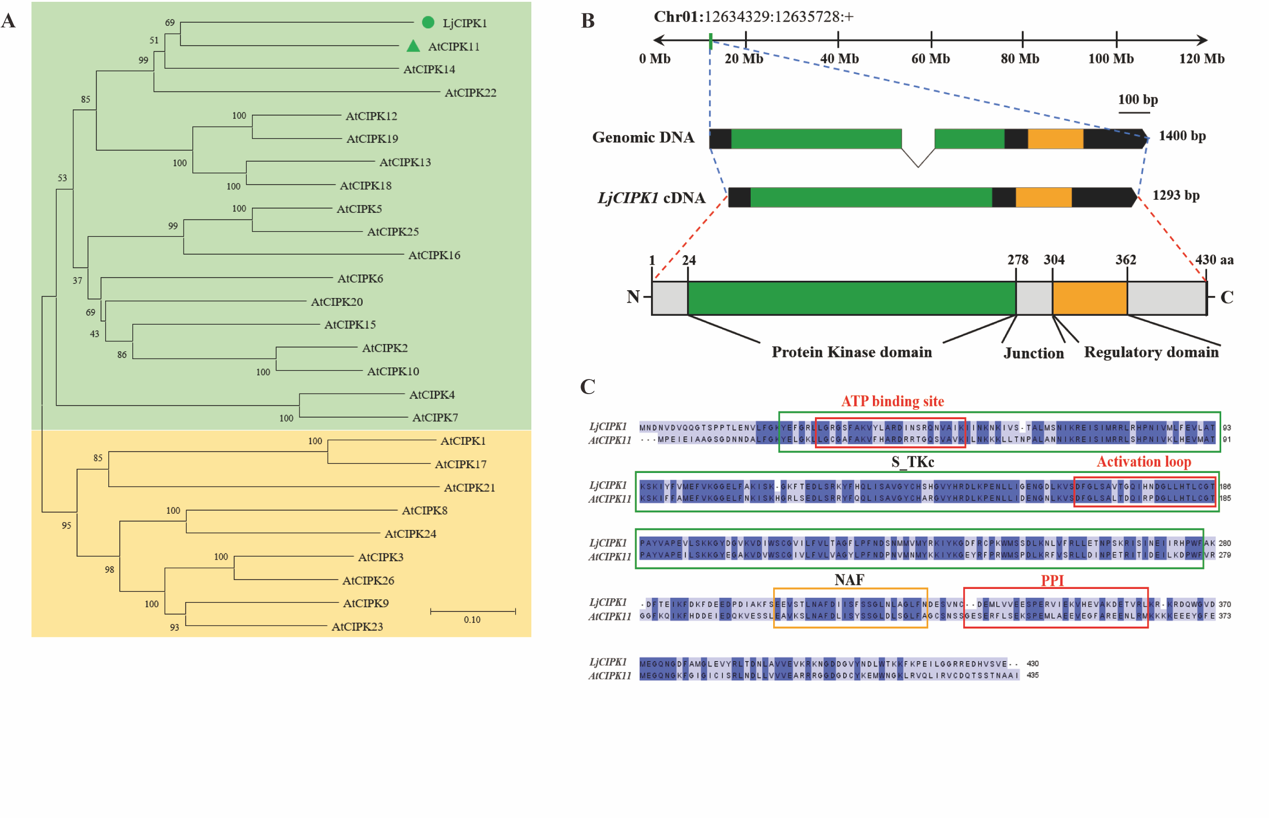
**

**Supplementary Figure 1.** Identification of *LjCIPK1* in *L. japonica.* (A) Phylogenetic tree of LjCIPK1 and CIPKs from *Arabidopsis* (At). (B) Genomic organization and chromosomal position of *LjCIPK1*. Exons (boxes) and introns (lines) are depicted, with their respective lengths (bp) labeled. Structural features were deduced by comparing genomic and complementary DNA sequences. (C) Comparative alignment of LjCIPK1 with AtCIPK11, highlighting conserved functional regions: Serine/Threonine kinase domain, NAF, activation loop, ATP-binding sites, and protein-protein interaction (PPI)


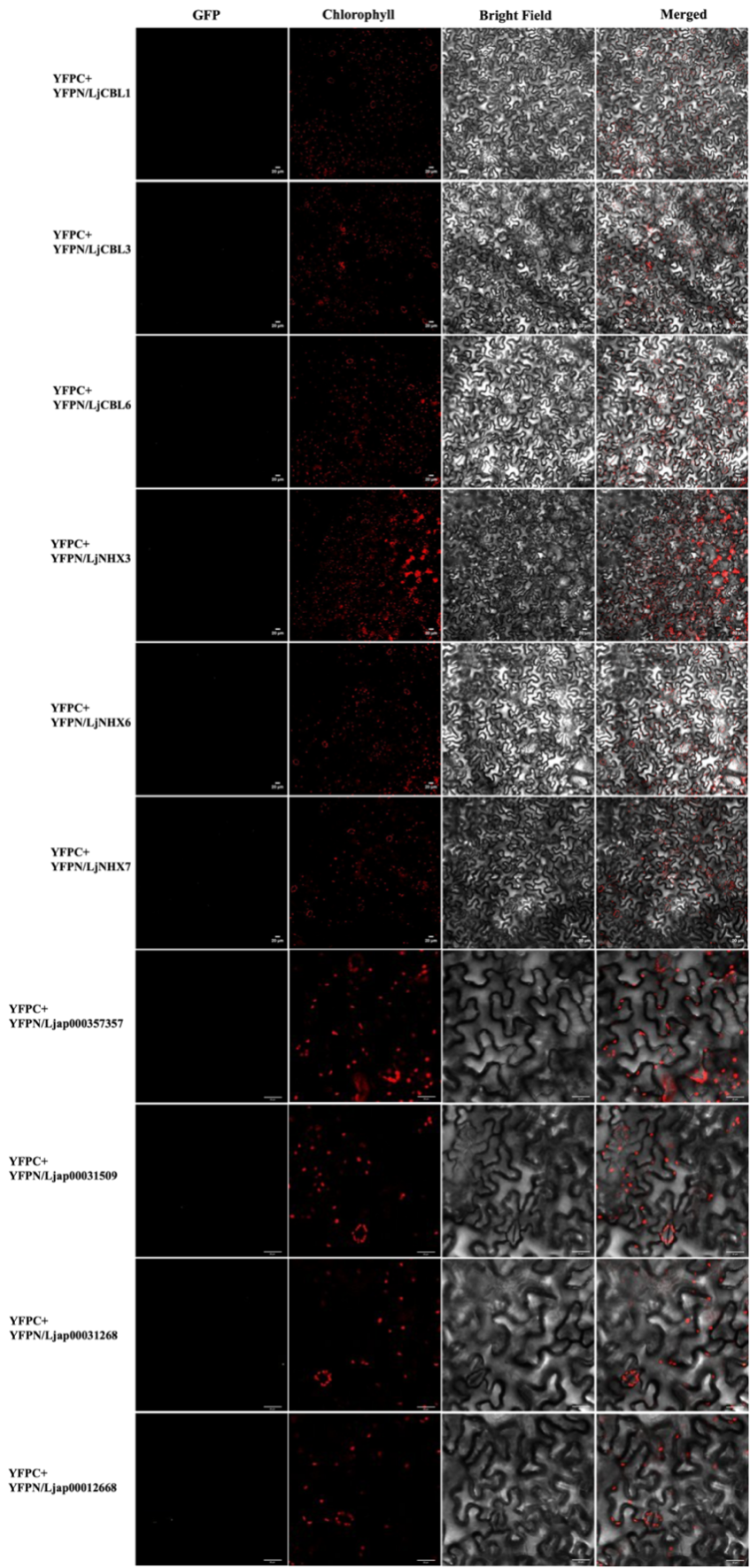


**Supplementary Figure 2.** BiFC negative control assays for LjCIPK1-interacting partners.


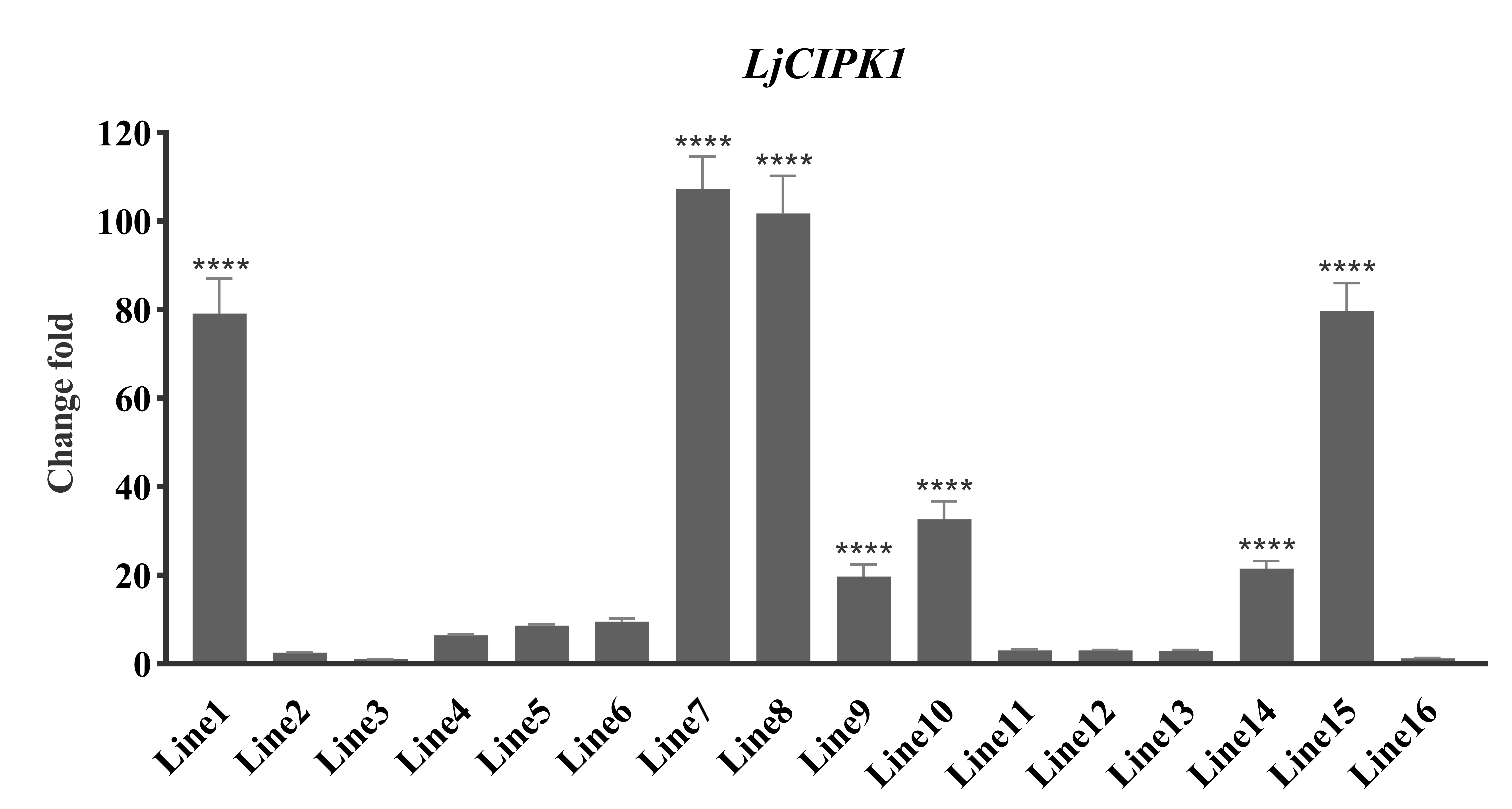


**Supplementary Figure 3.** qRT-PCR analysis of *LjCIPK1* expression in transgenic *Arabidopsis* lines.


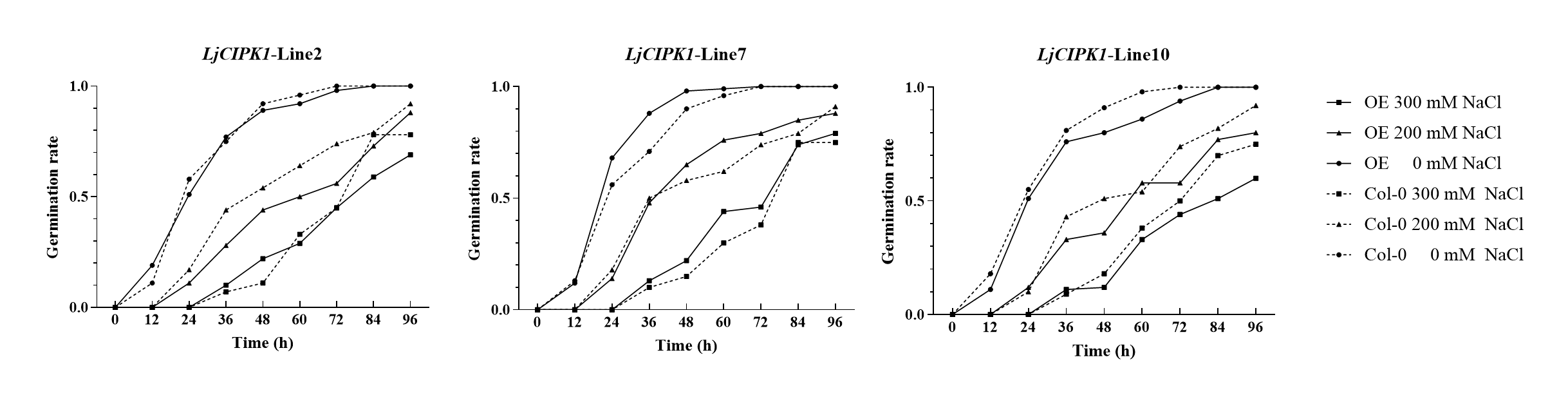


**Supplementary Figure 4.** Germination rates of transgenic *Arabidopsis* overexpressing of LjCIPK1 under mannitol stress.


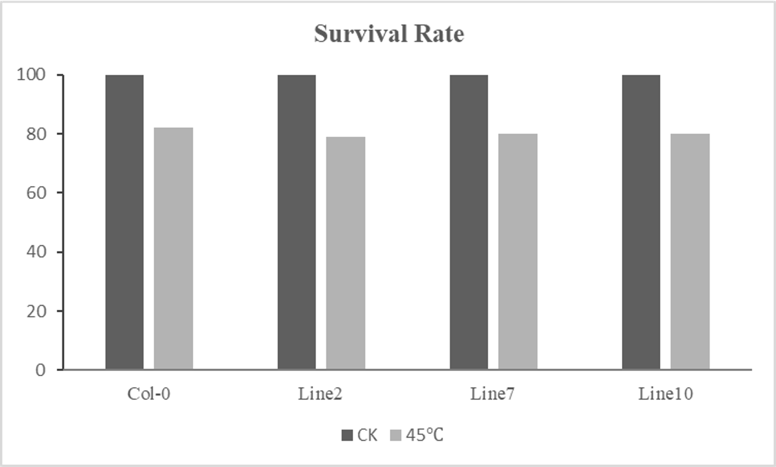


**Supplementary Figure 5.** Survival rates of transgenic *Arabidopsis* overexpressing of LjCIPK1 under heat stress.


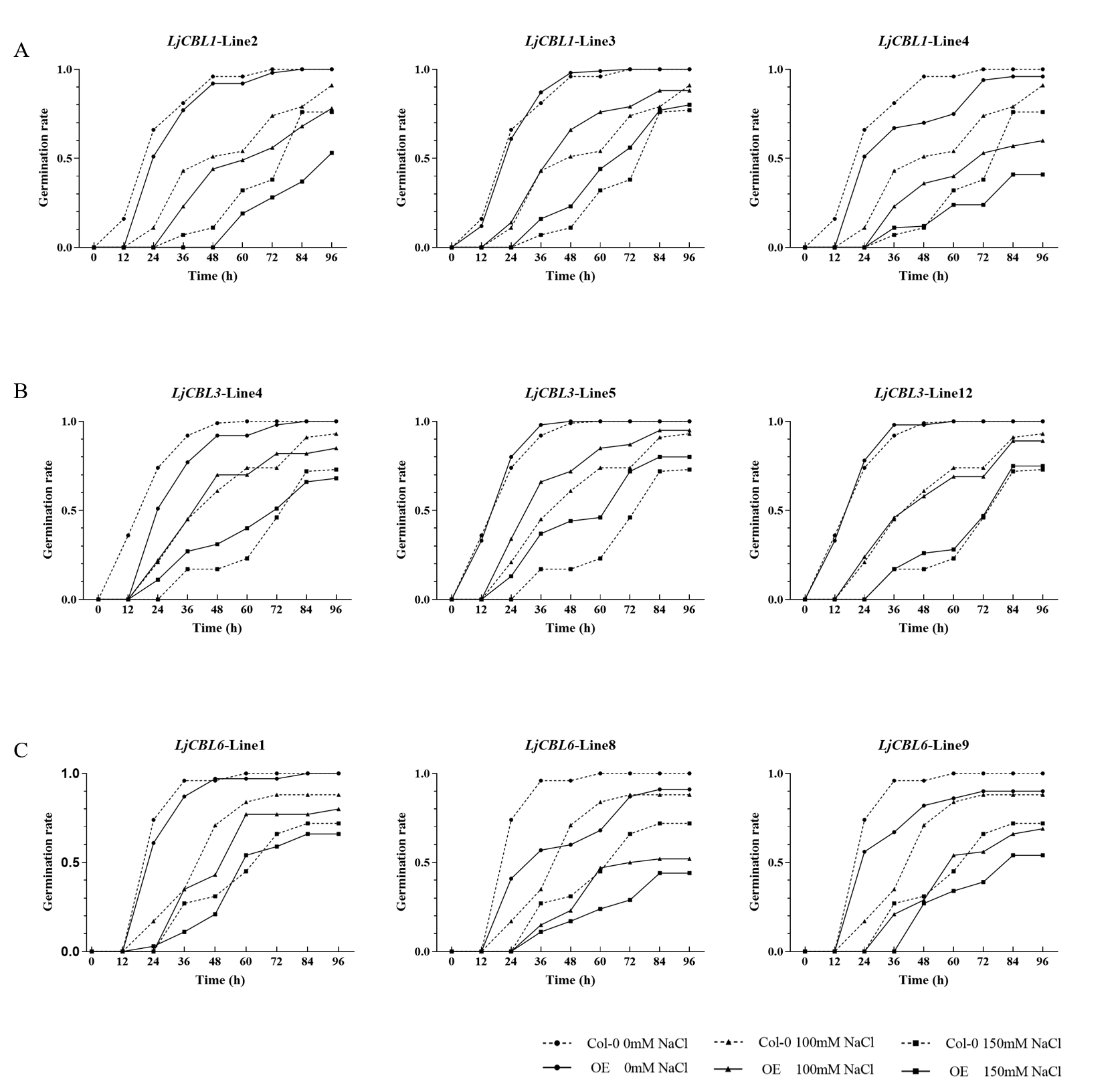


**Supplementary Figure 6.** Germination rates of transgenic *Arabidopsis* overexpressing of LjCBL1/3/6 under salt stress.

## Supplementary Tables

| **Supplementary Table 1.** List of the primer sequences | | |
| --- | --- | --- |
| *LjCIPK1*-OE | F | TGGAGAGAACACGGGGGACTCTAGAATGAACGACAATGTAGACGTGCA |
|  | R | GAACGATCGGGGAAATTCGAGCTCCCTACTCAACGGAAACATGATCCTC |
| *LjCIPK1*-GUS | F | AGCTCCTCGCCCTTGCTCACCATGGACATTGGTTAATCATATTAAATTGC |
|  | R | AAATTTACCCTCAGATCTACCATGGTGGAACGATGCGGAGATCGAA |
| *LjCIPK1*-SE | F | TCCACAATGACGGTCTGTTGC |
|  | R | CGCATGACCAAATATCCACCT |
| *TUB2*-RT | F | CTCAAGAGGTTCTCAGCAGTACC |
|  | R | TTTGTGCTCATCTTGCCACGGAAC |
| *LjCIPK1*-RT | F | ATCCACAATGACGGTCTG |
|  | R | AATATCCACCTTCACACCAT |
| *AtPAL*-RT | F | CGCTCTTCGTACTTCTCCTC |
|  | R | GCTATCGCCAATCTCGTGTT |
| *AtC4H*-RT | F | TGATTCTCGCCACGGTGATT |
|  | R | TGAGGAGACGACGACTAGGT |
| *At4C*L-RT | F | CTTGTACCGCCGCTAGTGAT |
|  | R | TCCTGCCTCCGTCATACCAT |
| *AtCHS*-RT | F | GTGATGGCTGGTGCTTCTTC |
|  | R | ACTGTTGGTGATGCGGAAGT |
| *AtCHI*-RT | F | CGCCGTTCCTTCTCTATCTG |
|  | R | CCACACAATTCTCCGTCACT |
| *AtFLS*-RT | F | ACTATTATCCGCCGTGTCCT |
|  | R | TCGCCGATGTGAACAATGAC |
| *AtDFR*-RT | F | CTTCGGGTTTCATCGGTTCAT |
|  | R | CATCGTAGCTTCCTTCCTCAG |
| *AtF3H*-RT | F | GTTGAGGCTTGTGAGAATTGG |
|  | R | CTGGAGGTGACTAGAGACGAT |
| *LjCIPK1*-GFP | F | TGGAGAGAACACGGGGGACTCTAGAATGTTGCAGTGCCTAGACGGATTAA |
|  | R | CATGGATCGAATTGATCCTCCTCCACCGGTATCGTCCACTTGGGAATGA |
| LjCIPK1-BD | F | CATATGGCCATGGAGGCCGAATTCATGAACGACAATGTAGACGTGCAGC |
|  | R | CGGCCGCTGCAGGTCGACGGATCCCTACTCAACGGAAACATGATCCTCC |
| LjCBL1-AD | F | TGGCCATGGAGGCCAGTGAATTCATGTTGCAGTGCCTAGACGGAT |
|  | R | TGCAGCTCGAGCTCGATGGATCCTCAGGTATCGTCCACTTGGGAAT |
| LjCBL2-AD | F | TGGCCATGGAGGCCAGTGAATTCATGGGTTATGCTTGCATGAA |
|  | R | TGCAGCTCGAGCTCGATGGATCCTCAATGATAGTACGTTGTTATAATC |
| LjCBL3-AD | F | TGGCCATGGAGGCCAGTGAATTCATGTTGCAGTGCTTAGAGGGG |
|  | R | TGCAGCTCGAGCTCGATGGATCCTCAAGTATCCTCAACTCTCGAGTG |
| LjCBL4-AD | F | TGGCCATGGAGGCCAGTGAATTCATGGGTTGTGCTTGCATG |
|  | R | TGCAGCTCGAGCTCGATGGATCCTTAATTCTTTTCAATGATAGTTGT |
| LjCBL5-AD | F | TGGCCATGGAGGCCAGTGAATTCATGGAATCCTCGACATCGAG |
|  | R | TGCAGCTCGAGCTCGATGGATCCTCAACCACAGTGTTGAAAATAAAAC |
| LjCBL6-AD | F | TGGCCATGGAGGCCAGTGAATTCATGGGCTGTTTTCAGTCTAAAGCAA |
|  | R | TGCAGCTCGAGCTCGATGGATCCTTAAGTAGCAAGCTCCTCAACCTC |
| LjNHX1-AD | F | TGGCCATGGAGGCCAGTGAATTCATGGGTTTCAGTTTTGGCTCTGTAT |
|  | R | TGCAGCTCGAGCTCGATGGATCCTTACTCGAGGGTTGTCGTTGCT |
| LjNHX2-AD | F | TGGCCATGGAGGCCAGTGAATTCATGGATACGTTATCTAATTCTGATC |
|  | R | TGCAGCTCGAGCTCGATGGATCCTTAGTGGGCCCCTTCTGATTG |
| LjNHX3-AD | F | TGGCCATGGAGGCCAGTGAATTCATGGCTTTCCATTTGATCAGTTCT |
|  | R | TGCAGCTCGAGCTCGATGGATCCTCACATCAATCCTGACCCTTCAG |
| LjNHX4-AD | F | TGGCCATGGAGGCCAGTGAATTCATGTTACTTTACCATTTTCCTTCAG |
|  | R | TGCAGCTCGAGCTCGATGGATCCTCAATTATCAGTTTCATTTGCAGC |
| LjNHX5-AD | F | TGGCCATGGAGGCCAGTGAATTCATGGGGTTTGATCTAGGATCTGTAG |
|  | R | TGCAGCTCGAGCTCGATGGATCCTCAATGCCACTGATTAACGTTGTTC |
| LjNHX6-AD | F | TGGCCATGGAGGCCAGTGAATTCATGGCATCTGTAGTAGGTGAAGGAG |
|  | R | TGCAGCTCGAGCTCGATGGATCCCTAGCGGAAAGAAAGCGTACTCGG |
| LjNHX7-AD | F | TGGCCATGGAGGCCAGTGAATTCATGAATACAATAACAACAAGTAGAG |
|  | R | TGCAGCTCGAGCTCGATGGATCCTCATGAGATCTCACAGATAGG |
| pGADT7 | T7 | TAATACGACTCACTATAGGGC |
|  | 3' AD | TGAGATGGTGCACGATGCACAGTTG |
| pGBKT7 | 3' BD | TAAGAGTCACTTTAAAAT TTGTATAC |
| Ljap00035735-AD | F | TGGCCATGGAGGCCAGTGAATTCATGGGAAGGTCTCCTTGCTG |
|  | R | TGCAGCTCGAGCTCGATGGATCCTCATTTCATCTCTAGGCTTCTG |
| Ljap00031509-AD | F | TGGCCATGGAGGCCAGTGAATTCATGGCTGGGATGTGCTG |
|  | R | CGAGCTCGATGGATCCCTATAGATCTCTCTTCAAATCAACC |
| Ljap00031268-AD | F | TGGCCATGGAGGCCAGTGAATTC ATGGTTCACCCCCACATCCT |
|  | R | TGCAGCTCGAGCTCGATGGATCCTTAGAGCTCTTCAACAAATTCTT |
| Ljap00012668-AD | F | TGGCCATGGAGGCCAGTGAATTCATGTTCTGCCTTTTCCAGGTTC |
|  | R | TGCAGCTCGAGCTCGATGGATCCTTACCTCTGTATGGACTTGTATAGT |

**Supplementary Table 2.** Protein sequences of LjCIPK1 and CIPKs from *Arabidopsis*.

| >LjCIPK1 |
| --- |
| MNDNVDVQQGTSPPTLENVLFGKYEFGRLLGRGSFAKVYLARDINSRQNVAIKIINKNKIVSTALMSNIKREISIMRRLRHPNIVMLFEVLATKSKIYFVMEFVKGGELFAKISKGKFTEDLSRKYFHQLISAVGYCHSHGVYHRDLKPENLLIGENGDLKVSDFGLSAVTGQIHNDGLLHTLCGTPAYVAPEVLSKKGYDGVKVDIWSCGVILFVLTAGFLPFNDSNMMVMYRKIYKGDFRCPKWMSSDLKNLVFRLLETNPSKRISINEIIRHPWFAKDFTEIKFDKFDEEDPDIAKFSEEVSTLNAFDIISFSSGLNLAGLFNDESVNCDEMLVVEESPERVIEKVHEVAKDETVRLKRKRDQWGVDMEGQNGDFAMGLEVYRLTDNLAVVEVKRKNGDDGVYNDLWTKKFKPEILGGRREDHVSVE |
| >AtCIPK1 |
| MVRRQEEEKKAEKGMRLGKYELGRTLGEGNFGKVKFAKDTVSGHSFAVKIIDKSRIADLNFSLQIKREIRTLKMLKHPHIVRLHEVLASKTKINMVMELVTGGELFDRIVSNGKLTETDGRKMFQQLIDGISYCHSKGVFHRDLKLENVLLDAKGHIKITDFGLSALPQHFRDDGLLHTTCGSPNYVAPEVLANRGYDGAASDIWSCGVILYVILTGCLPFDDRNLAVLYQKICKGDPPIPRWLSPGARTMIKRMLDPNPVTRITVVGIKASEWFKLEYIPSIPDDDDEEEVDTDDDAFSIQELGSEEGKGSDSPTIINAFQLIGMSSFLDLSGFFEQENVSERRIRFTSNSSAKDLLEKIETAVTEMGFSVQKKHAKLRVKQEERNQKGQVGLSVTAEVFEIKPSLNVVELRKSYGDSCLYRQLYERLLKDVGTSSPEQEIVT |
| >AtCIPK2 |
| MENKPSVLTERYEVGRLLGQGTFAKVYFGRSNHTNESVAIKMIDKDKVMRVGLSQQIKREISVMRIAKHPNVVELYEVMATKSRIYFVIEYCKGGELFNKVAKGKLKEDVAWKYFYQLISAVDFCHSRGVYHRDIKPENLLLDDNDNLKVSDFGLSALADCKRQDGLLHTTCGTPAYVAPEVINRKGYEGTKADIWSCGVVLFVLLAGYLPFHDTNLMEMYRKIGKADFKCPSWFAPEVKRLLCKMLDPNHETRITIAKIKESSWFRKGLHLKQKKMEKMEKQQVREATNPMEAGGSGQNENGENHEPPRLATLNAFDIIALSTGFGLAGLFGDVYDKRESRFASQKPASEIISKLVEVAKCLKLKIRKQGAGLFKLERVKEGKNGILTMDAEIFQVTPTFHLVEVKKCNGDTMEYQKLVEEDLRPALADIVWVWQGEKEKEEQLLQDEQGEQEPS |
| >AtCIPK3 |
| MLIPNKKLREMNRRQQVKRRVGKYEVGRTIGEGTFAKVKFARNSETGEPVALKILDKEKVLKHKMAEQIRREIATMKLIKHPNVVQLYEVMASKTKIFIILEYVTGGELFDKIVNDGRMKEDEARRYFQQLIHAVDYCHSRGVYHRDLKPENLLLDSYGNLKISDFGLSALSQQVRDDGLLHTSCGTPNYVAPEVLNDRGYDGATADMWSCGVVLYVLLAGYLPFDDSNLMNLYKKISSGEFNCPPWLSLGAMKLITRILDPNPMTRVTPQEVFEDEWFKKDYKPPVFEERDDSNMDDIDAVFKDSEEHLVTEKREEQPAAINAFEIISMSRGLNLENLFDPEQEFKRETRITLRGGANEIIEKIEEAAKPLGFDVQKKNYKMRLENVKAGRKGNLNVATEIFQVAPSLHMVQVSKSKGDTLEFHKFYKKLSNSLEQVVWTNNEVKKETAK |
| >AtCIPK4 |
| MESPYPKSPEKITGTVLLGKYELGRRLGSGSFAKVHVARSISTGELVAIKIIDKQKTIDSGMEPRIIREIEAMRRLHNHPNVLKIHEVMATKSKIYLVVEYAAGGELFTKLIRFGRLNESAARRYFQQLASALSFCHRDGIAHRDVKPQNLLLDKQGNLKVSDFGLSALPEHRSNNGLLHTACGTPAYTAPEVIAQRGYDGAKADAWSCGVFLFVLLAGYVPFDDANIVAMYRKIHKRDYRFPSWISKPARSIIYKLLDPNPETRMSIEAVMGTVWFQKSLEISEFQSSVFELDRFLEKEAKSSNAITAFDLISLSSGLDLSGLFERRKRKEKRFTARVSAERVVEKAGMIGEKLGFRVEKKEETKVVGLGKGRTAVVVEVVEFAEGLVVADVKVVVEGEEEEEEVESHWSELIVELEEIVLSWHN |
| >AtCIPK5 |
| MEEERRVLFGKYEMGRLLGKGTFAKVYYGKEIIGGECVAIKVINKDQVMKRPGMMEQIKREISIMKLVRHPNIVELKEVMATKTKIFFVMEFVKGGELFCKISKGKLHEDAARRYFQQLISAVDYCHSRGVSHRDLKPENLLLDENGDLKISDFGLSALPEQILQDGLLHTQCGTPAYVAPEVLKKKGYDGAKADIWSCGVVLYVLLAGCLPFQDENLMNMYRKIFRADFEFPPWFSPEARRLISKLLVVDPDRRISIPAIMRTPWLRKNFTPPLAFKIDEPICSQSSKNNEEEEEDGDCENQTEPISPKFFNAFEFISSMSSGFDLSSLFESKRKVQSVFTSRSSATEVMEKIETVTKEMNMKVKRTKDFKVKMEGKTEGRKGRLSMTAEVFEVAPEISVVEFCKSAGDTLEYDRLYEEEVRPALNDIVWSWHGDNNNTSSEDC |
| >AtCIPK6 |
| MVGAKPVENGSDGGSSTGLLHGRYELGRLLGHGTFAKVYHARNIQTGKSVAMKVVGKEKVVKVGMVDQIKREISVMRMVKHPNIVELHEVMASKSKIYFAMELVRGGELFAKVAKGRLREDVARVYFQQLISAVDFCHSRGVYHRDLKPENLLLDEEGNLKVTDFGLSAFTEHLKQDGLLHTTCGTPAYVAPEVILKKGYDGAKADLWSCGVILFVLLAGYLPFQDDNLVNMYRKIYRGDFKCPGWLSSDARRLVTKLLDPNPNTRITIEKVMDSPWFKKQATRSRNEPVAATITTTEEDVDFLVHKSKEETETLNAFHIIALSEGFDLSPLFEEKKKEEKREMRFATSRPASSVISSLEEAARVGNKFDVRKSESRVRIEGKQNGRKGKLAVEAEIFAVAPSFVVVEVKKDHGDTLEYNNFCSTALRPALKDIFWTSTPA |
| >AtCIPK7 |
| MESLPQPQNQSSPATTPAKILLGKYELGRRLGSGSFAKVHLARSIESDELVAVKIIEKKKTIESGMEPRIIREIDAMRRLRHHPNILKIHEVMATKSKIYLVMELASGGELFSKVLRRGRLPESTARRYFQQLASALRFSHQDGVAHRDVKPQNLLLDEQGNLKVSDFGLSALPEHLQNGLLHTACGTPAYTAPEVISRRGYDGAKADAWSCGVILFVLLVGDVPFDDSNIAAMYRKIHRRDYRFPSWISKQAKSIIYQMLDPNPVTRMSIETVMKTNWFKKSLETSEFHRNVFDSEVEMKSSVNSITAFDLISLSSGLDLSGLFEAKKKKERRFTAKVSGVEVEEKAKMIGEKLGYVVKKKMMKKEGEVKVVGLGRGRTVIVVEAVELTVDVVVVEVKVVEGEEDDSRWSDLITELEDIVLSWHNDIM |
| >AtCIPK8 |
| MVVRKVGKYELGRTIGEGTFAKVKFAQNTETGESVAMKIVDRSTIIKRKMVDQIKREISIMKLVRHPCVVRLYEVLASRTKIYIILEYITGGELFDKIVRNGRLSESEARKYFHQLIDGVDYCHSKGVYHRDLKPENLLLDSQGNLKISDFGLSALPEQGVTILKTTCGTPNYVAPEVLSHKGYNGAVADIWSCGVILYVLMAGYLPFDEMDLPTLYSKIDKAEFSCPSYFALGAKSLINRILDPNPETRITIAEIRKDEWFLKDYTPVQLIDYEHVNLDDVYAAFDDPEEQTYAQDGTRDTGPLTLNAFDLIILSQGLNLATLFDRGKDSMKHQTRFISHKPANVVLSSMEVVSQSMGFKTHIRNYKMRVEGLSANKTSHFSVILEVFKVAPSILMVDIQNAAGDAEEYLKFYKTFCSKLDDIIWKPPDASMRNRVTKAKSKRR |
| >AtCIPK9 |
| MSGSRRKATPASRTRVGNYEMGRTLGEGSFAKVKYAKNTVTGDQAAIKILDREKVFRHKMVEQLKREISTMKLIKHPNVVEIIEVMASKTKIYIVLELVNGGELFDKIAQQGRLKEDEARRYFQQLINAVDYCHSRGVYHRDLKPENLILDANGVLKVSDFGLSAFSRQVREDGLLHTACGTPNYVAPEVLSDKGYDGAAADVWSCGVILFVLMAGYLPFDEPNLMTLYKRICKAEFSCPPWFSQGAKRVIKRILEPNPITRISIAELLEDEWFKKGYKPPSFDQDDEDITIDDVDAAFSNSKECLVTEKKEKPVSMNAFELISSSSEFSLENLFEKQAQLVKKETRFTSQRSASEIMSKMEETAKPLGFNVRKDNYKIKMKGDKSGRKGQLSVATEVFEVAPSLHVVELRKTGGDTLEFHKVCDSFYKNFSSGLKDVVWNTDAAAEEQKQ |
| >AtCIPK10 |
| MENKPSVLTDKYDVGRLLGQGTFAKVYYGRSILTNQSVAIKMIDKEKVMKVGLIEQIKREISVMRIARHPNVVELYEVMATKTRIYFVMEYCKGGELFNKVAKGKLRDDVAWKYFYQLINAVDFCHSREVYHRDIKPENLLLDDNENLKVSDFGLSALADCKRQDGLLHTTCGTPAYVAPEVINRKGYDGTKADIWSCGVVLFVLLAGYLPFHDSNLMEMYRKIGKADFKAPSWFAPEVRRLLCKMLDPNPETRITIARIRESSWFRKGLHMKQKKMEKRVKEINSVEAGTAGTNENGAGPSENGAGPSENGDRVTEENHTDEPTNLNAFDLIALSAGFDLAGLFGDDNKRESRFTSQKPASVIISKLEEVAQRLKLSIRKREAGLFKLERLKEGRKGILSMDAEIFQVTPNFHLVEVKKSNGDTLEYQKLVAEDLRPALSDIVWVWQGEKDELTSQQETEYQQQQQQEQQEQEEPLKF |
| >AtCIPK11 |
| MPEIEIAAGSGDNNDALFGKYELGKLLGCGAFAKVFHARDRRTGQSVAVKILNKKKLLTNPALANNIKREISIMRRLSHPNIVKLHEVMATKSKIFFAMEFVKGGELFNKISKHGRLSEDLSRRYFQQLISAVGYCHARGVYHRDLKPENLLIDENGNLKVSDFGLSALTDQIRPDGLLHTLCGTPAYVAPEILSKKGYEGAKVDVWSCGIVLFVLVAGYLPFNDPNVMNMYKKIYKGEYRFPRWMSPDLKRFVSRLLDINPETRITIDEILKDPWFVRGGFKQIKFHDDEIEDQKVESSLEAVKSLNAFDLISYSSGLDLSGLFAGCSNSSGESERFLSEKSPEMLAEEVEGFAREENLRMKKKKEEEYGFEMEGQNGKFGIGICISRLNDLLVVVEARRRGGDGDCYKEMWNGKLRVQLIRVCDQTSSTNAAI |
| >AtCIPK12 |
| MAEKITRETSLPKERSSPQALILGRYEMGKLLGHGTFAKVYLARNVKTNESVAIKVIDKEKVLKGGLIAHIKREISILRRVRHPNIVQLFEVMATKAKIYFVMEYVRGGELFNKVAKGRLKEEVARKYFQQLISAVTFCHARGVYHRDLKPENLLLDENGNLKVSDFGLSAVSDQIRQDGLFHTFCGTPAYVAPEVLARKGYDAAKVDIWSCGVILFVLMAGYLPFHDRNVMAMYKKIYRGEFRCPRWFSTELTRLLSKLLETNPEKRFTFPEIMENSWFKKGFKHIKFYVEDDKLCNVVDDDELESDSVESDRDSAASESEIEYLEPRRRVGGLPRPASLNAFDIISFSQGFDLSGLFDDDGEGSRFVSGAPVSKIISKLEEIAKVVSFTVRKKDCRVSLEGSRQGVKGPLTIAAEIFELTPSLVVVEVKKKGGDKTEYEDFCNNELKPKLQNLTADDVVAEPVAVSAVDETAIPNSPTISFLPSDTE |
| >AtCIPK13 |
| MAQVLSTPLAIPGPTPIQFMAGLLARIVTKNTNKETSTPESPRSPRTPQGSILMDKYEIGKLLGHGSFAKVYLARNIHSGEDVAIKVIDKEKIVKSGLAGHIKREISILRRVRHPYIVHLLEVMATKTKIYIVMEYVRGGELYNTVARGRLREGTARRYFQQLISSVAFCHSRGVYHRDLKLENLLLDDKGNVKVSDFGLSVVSEQLKQEGICQTFCGTPAYLAPEVLTRKGYEGAKADIWSCGVILFVLMAGYLPFDDKNILVMYTKIYKGQFKCPKWFSPELARLVTRMLDTNPDTRITIPEIMKHRWFKKGFKHVKFYIENDKLCREDDDNDDDDSSSLSSGRSSTASEGDAEFDIKRVDSMPRPASLNAFDILSFSDLSGLFEEGGQGARFVSAAPMTKIISKLEEIAKEVKFMVRKKDWSVRLEGCREGAKGPLTIRVEIFELTPSLVVVEVKKKGGNIEEYEEFCNKELRPQLEKLMHYQADEVEEVMCLPPEIEQ |
| >AtCIPK14 |
| MVDSDPVEFPPENRRGQLFGKYEVGKLVGCGAFAKVYHGRSTATGQSVAIKVVSKQRLQKGGLNGNIQREIAIMHRLRHPSIVRLFEVLATKSKIFFVMEFAKGGELFAKVSKGRFCEDLSRRYFQQLISAVGYCHSRGIFHRDLKPENLLLDEKLDLKISDFGLSALTDQIRPDGLLHTLCGTPAYVAPEVLAKKGYDGAKIDIWSCGIILFVLNAGYLPFNDHNLMVMYRKIYKGEFRIPKWTSPDLRRLLTRLLDTNPQTRITIEEIIHDPWFKQGYDDRMSKFHLEDSDMKLPADETDSEMGARRMNAFDIISGSPGFNLSGLFGDARKYDRVERFVSAWTAERVVERLEEIVSAENLTVAKKETWGMKIEGQKGNFAMVVEINQLTDELVMIEVRKRQRAAASGRDLWTDTLRPFFVELVHESDQTDPEPTQVHTTS |
| >AtCIPK15 |
| MEKKGSVLMLRYEVGKFLGQGTFAKVYHARHLKTGDSVAIKVIDKERILKVGMTEQIKREISAMRLLRHPNIVELHEVMATKSKIYFVMEHVKGGELFNKVSTGKLREDVARKYFQQLVRAVDFCHSRGVCHRDLKPENLLLDEHGNLKISDFGLSALSDSRRQDGLLHTTCGTPAYVAPEVISRNGYDGFKADVWSCGVILFVLLAGYLPFRDSNLMELYKKIGKAEVKFPNWLAPGAKRLLKRILDPNPNTRVSTEKIMKSSWFRKGLQEEVKESVEEETEVDAEAEGNASAEKEKKRCINLNAFEIISLSTGFDLSGLFEKGEEKEEMRFTSNREASEITEKLVEIGKDLKMKVRKKEHEWRVKMSAEATVVEAEVFEIAPSYHMVVLKKSGGDTAEYKRVMKESIRPALIDFVLAWH |
| >AtCIPK16 |
| MEESNRSSTVLFDKYNIGRLLGTGNFAKVYHGTEISTGDDVAIKVIKKDHVFKRRGMMEQIEREIAVMRLLRHPNVVELREVMATKKKIFFVMEYVNGGELFEMIDRDGKLPEDLARKYFQQLISAVDFCHSRGVFHRDIKPENLLLDGEGDLKVTDFGLSALMMPEGLGGRRGSSDDLLHTRCGTPAYVAPEVLRNKGYDGAMADIWSCGIVLYALLAGFLPFIDENVMTLYTKIFKAECEFPPWFSLESKELLSRLLVPDPEQRISMSEIKMIPWFRKNFTPSVAFSIDETIPSPPEPPTKKKKKDLNEKEDDGASPRSFNAFQFITSMSSGFDLSNLFEIKRKPKRMFTSKFPAKSVKERLETAAREMDMRVKHVKDCKMKLQRRTEGRKGRLSVTAEVFEVAPEVSVVEFCKTSGDTLEYYLFCEDDVRPALKDIVWSWQGDDDEDDVTTNDNVDTNDNKINNVS |
| >AtCIPK17 |
| MVIKGMRVGKYELGRTLGEGNSAKVKFAIDTLTGESFAIKIIEKSCITRLNVSFQIKREIRTLKVLKHPNIVRLHEVLASKTKIYMVLECVTGGDLFDRIVSKGKLSETQGRKMFQQLIDGVSYCHNKGVFHRDLKLENVLLDAKGHIKITDFGLSALSQHYREDGLLHTTCGSPNYVAPEVLANEGYDGAASDIWSCGVILYVILTGCLPFDDANLAVICRKIFKGDPPIPRWISLGAKTMIKRMLDPNPVTRVTIAGIKAHDWFKHDYTPSNYDDDDDVYLIQEDVFMMKEYEEEKSPDSPTIINAFQLIGMSSFLDLSGFFETEKLSERQIRFTSNSLAKDLLENIETIFTEMGFCLQKKHAKLKAIKEESTQKRQCGLSVTAEVFEISPSLNVVELRKSHGDSSLYKQLYERLLNELGSSSQVQELLA |
| >AtCIPK18 |
| MAQALAQPPLVVTTVVPDPPPPPPPPHPKPYALRYMADLLGRIGIMDTDKDGNISPQSPRSPRSPRNNILMGKYELGKLLGHGTFAKVYLAQNIKSGDKVAIKVIDKEKIMKSGLVAHIKREISILRRVRHPYIVHLFEVMATKSKIYFVMEYVGGGELFNTVAKGRLPEETARRYFQQLISSVSFCHGRGVYHRDLKPENLLLDNKGNLKVSDFGLSAVAEQLRQDGLCHTFCGTPAYIAPEVLTRKGYDAAKADVWSCGVILFVLMAGHIPFYDKNIMVMYKKIYKGEFRCPRWFSSDLVRLLTRLLDTNPDTRITIPEIMKNRWFKKGFKHVKFYIEDDKLCREDEDEEEEASSSGRSSTVSESDAEFDVKRMGIGSMPRPSSLNAFDIISFSSGFDLSGLFEEEGGEGTRFVSGAPVSKIISKLEEIAKIVSFTVRKKEWSLRLEGCREGAKGPLTIAAEIFELTPSLVVVEVKKKGGDREEYEEFCNKELRPELEKLIHEEVVVEEALYLPSDTE |
| >AtCIPK19 |
| MADLLRKVKSIKKKQDQSNHQALILGKYEMGRLLGHGTFAKVYLARNAQSGESVAIKVIDKEKVLKSGLIAHIKREISILRRVRHPNIVQLFEVMATKSKIYFVMEYVKGGELFNKVAKGRLKEEMARKYFQQLISAVSFCHFRGVYHRDLKPENLLLDENGNLKVSDFGLSAVSDQIRQDGLFHTFCGTPAYVAPEVLARKGYDGAKVDIWSCGVILFVLMAGFLPFHDRNVMAMYKKIYRGDFRCPRWFPVEINRLLIRMLETKPERRFTMPDIMETSWFKKGFKHIKFYVEDDHQLCNVADDDEIESIESVSGRSSTVSEPEDFESFDGRRRGGSMPRPASLNAFDLISFSPGFDLSGLFEDDGEGSRFVSGAPVGQIISKLEEIARIVSFTVRKKDCKVSLEGSREGSMKGPLSIAAEIFELTPALVVVEVKKKGGDKMEYDEFCNKELKPKLQNLSSENGQRVSGSRSLPSFLLSDTD |
| >AtCIPK20 |
| MDKNGIVLMRKYELGRLLGQGTFAKVYHARNIKTGESVAIKVIDKQKVAKVGLIDQIKREISVMRLVRHPHVVFLHEVMASKTKIYFAMEYVKGGELFDKVSKGKLKENIARKYFQQLIGAIDYCHSRGVYHRDLKPENLLLDENGDLKISDFGLSALRESKQQDGLLHTTCGTPAYVAPEVIGKKGYDGAKADVWSCGVVLYVLLAGFLPFHEQNLVEMYRKITKGEFKCPNWFPPEVKKLLSRILDPNPNSRIKIEKIMENSWFQKGFKKIETPKSPESHQIDSLISDVHAAFSVKPMSYNAFDLISSLSQGFDLSGLFEKEERSESKFTTKKDAKEIVSKFEEIATSSERFNLTKSDVGVKMEDKREGRKGHLAIDVEIFEVTNSFHMVEFKKSGGDTMEYKQFCDRELRPSLKDIVWKWQGNNNNSNNEKIEVIH |
| >AtCIPK21 |
| MGLFGTKKIGKYEIGRTIGEGNFAKVKLGYDTTNGTYVAVKIIDKALVIQKGLESQVKREIRTMKLLNHPNIVQIHEVIGTKTKICIVMEYVSGGQLSDRLGRQKMKESDARKLFQQLIDAVDYCHNRGVYHRDLKPQNLLLDSKGNLKVSDFGLSAVPKSGDMLSTACGSPCYIAPELIMNKGYSGAAVDVWSCGVILFELLAGYPPFDDHTLPVLYKKILRADYTFPPGFTGEQKRLIFNILDPNPLSRITLAEIIIKDSWFKIGYTPVYHQLSDSIKDNVAEINAATASSNFINAFQIIAMSSDLDLSGLFEENDDKRYKTRIGSKNTAQETIKKIEAAATYVSLSVERIKHFKVKIQPKEIRSRSSYDLLSAEVIEVTPTNCVIEISKSAGELRLYMEFCQSLSSLLTAEVS |
| >AtCIPK22 |
| MAEDSNSSESIIVNVTGDDNKSALFGKYDLGKLLGSGAFAKVYQAEDLQNGGESVAIKVVQKKRLKDGLTAHVKREISVMRRLRHPHIVLLSEVLATKTKIYFVMELAKGGELFSRVTSNRFTESLSRKYFRQLISAVRYCHARGVFHRDLKPENLLLDENRDLKVSDFGLSAMKEQIHPDGMLHTLCGTPAYVAPELLLKKGYDGSKADIWSCGVVLFLLNAGYLPFRDPNIMGLYRKIHKAQYKLPDWTSSDLRKLLRRLLEPNPELRITVEEILKDPWFNHGVDPSEIIGIQADDYDLEENGKILNAFDLISSASSSNLSGLFGNFVTPDHCDQFVSDESTAVIMRKVEEVAKQLNLRIAKKKERAIKLEGPHGVANVVVKVRRLTNELVMVEMKNKQRDVGLVWADALRQKLRRLINQPVYKVPDKP |
| >AtCIPK23 |
| MASRTTPSRSTPSRSTPSGSSSGGRTRVGKYELGRTLGEGTFAKVKFARNVENGDNVAIKVIDKEKVLKNKMIAQIKREISTMKLIKHPNVIRMFEVMASKTKIYFVLEFVTGGELFDKISSNGRLKEDEARKYFQQLINAVDYCHSRGVYHRDLKPENLLLDANGALKVSDFGLSALPQQVREDGLLHTTCGTPNYVAPEVINNKGYDGAKADLWSCGVILFVLMAGYLPFEDSNLTSLYKKIFKAEFTCPPWFSASAKKLIKRILDPNPATRITFAEVIENEWFKKGYKAPKFENADVSLDDVDAIFDDSGESKNLVVERREEGLKTPVTMNAFELISTSQGLNLGSLFEKQMGLVKRKTRFTSKSSANEIVTKIEAAAAPMGFDVKTNNYKMKLTGEKSGRKGQLAVATEVFQVAPSLYMVEMRKSGGDTLEFHKFYKNLTTGLKDIVWKTIDEEKEEGTDGGGTNGAMANRTIAKQST |
| >AtCIPK24 |
| MTKKMRRVGKYEVGRTIGEGTFAKVKFARNTDTGDNVAIKIMAKSTILKNRMVDQIKREISIMKIVRHPNIVRLYEVLASPSKIYIVLEFVTGGELFDRIVHKGRLEESESRKYFQQLVDAVAHCHCKGVYHRDLKPENLLLDTNGNLKVSDFGLSALPQEGVELLRTTCGTPNYVAPEVLSGQGYDGSAADIWSCGVILFVILAGYLPFSETDLPGLYRKINAAEFSCPPWFSAEVKFLIHRILDPNPKTRIQIQGIKKDPWFRLNYVPIRAREEEEVNLDDIRAVFDGIEGSYVAENVERNDEGPLMMNAFEMITLSQGLNLSALFDRRQDFVKRQTRFVSRREPSEIIANIEAVANSMGFKSHTRNFKTRLEGLSSIKAGQLAVVIEIYEVAPSLFMVDVRKAAGETLEYHKFYKKLCSKLENIIWRATEGIPKSEILRTITF |
| >AtCIPK25 |
| MGSKLKLYPLLNHSSVFHPDSRYQSAPTMEEEQQQLRVLFAKYEMGRLLGKGTFGKVYYGKEITTGESVAIKIINKDQVKREGMMEQIKREISIMRLVRHPNIVELKEVMATKTKIFFIMEYVKGGELFSKIVKGKLKEDSARKYFQQLISAVDFCHSRGVSHRDLKPENLLVDENGDLKVSDFGLSALPEQILQDGLLHTQCGTPAYVAPEVLRKKGYDGAKGDIWSCGIILYVLLAGFLPFQDENLMKMYRKIFKSEFEYPPWFSPESKRLISKLLVVDPNKRISIPAIMRTPWFRKNINSPIEFKIDELEIQNVEDETPTTTATTATTTTTPVSPKFFNAFEFISSMSSGFDLSSLFESKRKLRSMFTSRWSASEIMGKLEGIGKEMNMKVKRTKDFKVKLFGKTEGRKGQIAVTAEVFEVAPEVAVVELCKSAGDTLEYNRLYEEHVRPALEEIVWSWHGDNHNNNIVKSNGNYVSDENSGSDC |
| >AtCIPK26 |
| MNRPKVQRRVGKYEVGKTLGQGTFAKVRCAVNTETGERVALKILDKEKVLKHKMAEQIRREICTMKLINHPNVVRLYEVLASKTKIYIVLEFGTGGELFDKIVHDGRLKEENARKYFQQLINAVDYCHSRGVYHRDLKPENLLLDAQGNLKVSDFGLSALSRQVRGDGLLHTACGTPNYAAPEVLNDQGYDGATADLWSCGVILFVLLAGYLPFEDSNLMTLYKKIIAGEYHCPPWLSPGAKNLIVRILDPNPMTRITIPEVLGDAWFKKNYKPAVFEEKEEANLDDVDAVFKDSEEHHVTEKKEEQPTSMNAFELISMSRALDLGNLFEEEEGFKRETRFAAKGAANDLVQKIEEASKPLGFDIQKKNYKMRLENVTAGRKGNLRVATEIFQVSPSLHMIEVRKTKGDTLEFHKFYKKLSTSLNDVVWKSGESSGLSK |

| **Supplementary Table 3.** Blast results of LjCIPK1 candidate interaction protein | | | | | | | | | |
| --- | --- | --- | --- | --- | --- | --- | --- | --- | --- |
| **ID** | **Description** | **Scientific Name** | **Max Score** | **Total Score** | **Query Cover** | **E-value** | **Per. Ident** | **Acc. Len** | **Accession** |
| Ljap00006106 | probable GTP-binding protein OBGC2 | *Actinidia eriantha* | 944 | 944 | 71% | 0 | 83.33% | 1808 | XM_057646356.1 |
| Ljap00007347 | protein Actinidia eriantha protein YIP4b-like | *Actinidia eriantha* | 662 | 662 | 100% | 0 | 81.33% | 1257 | XM_057631797.1 |
| Ljap00007719 | uncharacterized protein LOC105163443 | *Solanum stenotomum* | 63.9 | 63.9 | 5% | 5.00E-05 | 93.02% | 916 | XM_049528472.1 |
| Ljap00008700 | E3 ubiquitin-protein ligase RGLG2 | *Cornus florida* | 1105 | 1105 | 85% | 0 | 83.74% | 1995 | XM_059784616.1 |
| Ljap00009573 | Cac030 AP2/ERF transcription factor mRNA | *Camptotheca acuminata* | 1026 | 1026 | 43% | 0 | 83.10% | 2640 | [MN863567.1](https://www.ncbi.nlm.nih.gov/nucleotide/MN863567.1?report=genbank&log$=nucltop&blast_rank=1&RID=R5SBXK11016) |
| Ljap00012608 | imidazoleglycerol-phosphate dehydratase, chloroplastic-like, transcript variant X1 | *Camellia sinensis* | 725 | 725 | 88% | 0 | 83.69% | 1301 | XM_028220624.1 |
| Ljap00012668 | SNF1-related protein kinase regulatory subunit beta-2-like | *Actinidia eriantha* | 667 | 667 | 98% | 0 | 84.72% | 1437 | XM_057643496.1 |
| Ljap00025360 | ubiquitin-conjugating enzyme 15, mRNA | *Cornus florida* | 279 | 279 | 36% | 3.00E-70 | 90.52% | 916 | XM_059781104.1 |
| Ljap00027354 | SNF1-related protein kinase catalytic subunit alpha KIN10 isoform X2 | *Cornus florida* | 1555 | 1555 | 77% | 0 | 88.54% | 2359 | XM_059814396.1 |
| Ljap00027488 | U-box domain-containing protein 4-like, transcript variant X5 | *Diospyros lotus* | 824 | 1259 | 77% | 0 | 80.58% | 3364 | XM_052340304.1 |
| Ljap00028523 | probable ADP-ribosylation factor GTPase-activating protein AGD14 isoform X2 | *Camellia sinensis* | 804 | 804 | 56% | 0 | 79.66% | 2945 | XM_028208475.1 |
| Ljap00029248 | isocitrate dehydrogenase [NADP] | *Cornus florida* | 1267 | 1267 | 92% | 0 | 86.18% | 1738 | XM_059767284.1 |
| Ljap00031268 | crocetin glucosyltransferase, chloroplastic-like | *Primulina huaijiensis* | 241 | 241 | 31% | 3.00E-58 | 77.37% | 1520 | XM_073443569.1 |
| Ljap00031509 | protein phosphatase 2C 37-like | *Camellia sinensis* | 499 | 499 | 64% | 5.00E-136 | 78.33% | 1657 | XM_028224989.1 |
| Ljap00032872 | probable alpha-mannosidase At5g13980 | *Actinidia eriantha* | 1170 | 2522 | 87% | 0 | 85.70% | 3747 | XM_057609109.1 |
| Ljap00035425 | uncharacterized LOC114318067, transcript variant X1 | *Camellia sinensis* | 933 | 933 | 81% | 0 | 85.71% | 1423 | XM_028264931.1 |
| Ljap00035735 | MYB42 protein (MYB42) mRNA | *Camellia japonica* | 544 | 544 | 100% | 1.00E-149 | 80.29% | 735 | PP033339.1 |
